# Genome-Wide Association for Sensitivity to Chronic Oxidative Stress in *Drosophila melanogaster*

**DOI:** 10.1371/journal.pone.0038722

**Published:** 2012-06-08

**Authors:** Katherine W. Jordan, Kyle L. Craver, Michael M. Magwire, Carmen E. Cubilla, Trudy F. C. Mackay, Robert R. H. Anholt

**Affiliations:** 1 Department of Genetics, North Carolina State University, Raleigh, North Carolina, United States of America; 2 W.M. Keck Center for Behavioral Biology, North Carolina State University, Raleigh, North Carolina, United States of America; 3 Department of Biology, North Carolina State University, Raleigh, North Carolina, United States of America; Semmelweis University, Hungary

## Abstract

Reactive oxygen species (ROS) are a common byproduct of mitochondrial energy metabolism, and can also be induced by exogenous sources, including UV light, radiation, and environmental toxins. ROS generation is essential for maintaining homeostasis by triggering cellular signaling pathways and host defense mechanisms. However, an imbalance of ROS induces oxidative stress and cellular death and is associated with human disease, including age-related locomotor impairment. To identify genes affecting sensitivity and resistance to ROS-induced locomotor decline, we assessed locomotion of aged flies of the sequenced, wild-derived lines from the *Drosophila melanogaster* Genetics Reference Panel on standard medium and following chronic exposure to medium supplemented with 3 mM menadione sodium bisulfite (MSB). We found substantial genetic variation in sensitivity to oxidative stress with respect to locomotor phenotypes. We performed genome-wide association analyses to identify candidate genes associated with variation in sensitivity to ROS-induced decline in locomotor performance, and confirmed the effects for 13 of 16 mutations tested in these candidate genes. Candidate genes associated with variation in sensitivity to MSB-induced oxidative stress form networks of genes involved in neural development, immunity, and signal transduction. Many of these genes have human orthologs, highlighting the utility of genome-wide association in Drosophila for studying complex human disease.

## Introduction

The production of free radicals is an inevitable consequence of aerobic life [1]–[3]. Reactive oxygen species (ROS) are a byproduct of mitochondrial energy metabolism, and can also be induced by exogenous sources, such as cytokines, UV light, radiation, and environmental toxins [1], [2]. The generation of ROS within certain boundaries is essential for maintaining homeostasis by triggering cellular signaling pathways and host defense mechanisms [1]. However, an imbalance in intracellular ROS can cause cellular damage [1], [4]. ROS have been implicated in aging [1], [3] and cardiovascular disease [4]–[6], stroke [7], and diabetes [8]. Oxidative stress is thought to contribute to neuronal cell death associated with Alzheimer's disease, Parkinson's disease, and amyotrophic lateral sclerosis [reviewed in 4].

Cellular defense mechanisms against oxidative stress include enzymatic antioxidants *Superoxide dismutase* (*Sod*), *Catalase* (*Cat*), *Glutathione reductase* (*GSR*), and *Glutathione peroxidase* (*GPx*) as ROS scavengers [1]. These enzymes modulate oxidative stress balance [9]–[11] and may contribute to the correlation between resistance to oxidative stress and lifespan [12]–[15]. Alleles of *age-1* in *Caenorhabditis elegans* [16], *methuselah* (*mth*) in Drosophila [17], and *shc^66^* in mice [18] are long-lived and resistant to oxidative stress. Dietary antioxidants, such as vitamin C, vitamin D, melatonin, and polyphenols ameliorate the effects of oxidative stress-inducing chemicals, such as paraquat, on Drosophila lifespan [19], [20], in a sexually dimorphic manner, with females exhibiting greater increases in lifespan [21].

Chronic exposure of flies to rotenone [22] and paraquat [23]–[25] produces dopaminergic cell death and locomotor deficits associated with oxidative stress. Such chronic exposure serves as a model for pesticide-induced Parkinson's disease. Genome-wide expression studies in flies following chemically induced oxidative stress have identified several genes with altered transcript abundances, but their identities depend on the nature of the agent that induces the oxidative stress [26]–[28]. In the present study, we used menadione sodium bisulfite (MSB), rather than paraquat or H_2_O_2_, which have commonly been used as agents to induce acute oxidative stress. MSB is milder, persists stably in the growth medium for prolonged time periods, and is effective in inducing chronic oxidative stress in adult flies during a two week exposure period, more closely mimicking human exposure. MSB at low concentrations mimics oxidant signaling and at higher concentrations induces lethal oxidant stress in cells from mice and chickens [29]. Accumulation of cholesterol aggravates MSB-induced oxidative stress and exacerbates apoptotic cell death [30] in wild type Chinese Hamster ovary cells. A genetic deletion of *PARP-1* confers protection from MSB-induced cell death [29], suggesting that both genetic variants and environment can increase resistance or susceptibility to oxidative stress.

Here, we capitalized on natural variation in the *Drosophila melanogaster* Genetic Reference Panel (DGRP) [31] to identify novel loci and a cellular network associated with variation in susceptibility to chronic MSB-induced oxidative stress, as measured by variation in locomotor impairments. Evolutionary conservation of the cellular pathways identified in this study can provide a blueprint for translational studies on the identification of human genetic risk factors associated with oxidative stress-related neurodegenerative diseases.

## Materials and Methods

### Drosophila culture

Flies from 192 inbred lines of the DGRP [31] were reared on standard cornmeal molasses agar medium, or on medium supplemented with MSB (Sigma-Aldrich M5750) at 25°C, 70% humidity, 12 hour light/dark cycle, and controlled density.

### Optimal MSB concentration

Five DGRP lines (RAL_304, RAL_313, RAL_517, RAL_732, RAL_852) were used to assess the effects of different concentrations of MSB on lifespan [32] using 12 replicates of 3 males and 3 females each. Flies were transferred to new vials every other day and the number of dead flies was recorded until all were dead. Tukey tests were performed to assess differences in average lifespan among MSB concentrations to determine an effective and maximally discriminating concentration of chronic MSB that shortens lifespan, but allows survival for at least 14 days to assess locomotor impairments.

### Startle response assay

Startle response was assessed as described previously [32], [33]. Briefly, single 13–16 day old flies, collected under CO_2_ exposure into vials containing 5 ml of standard cornmeal-agar-molasses medium, were left overnight. Startle responses were quantified the following morning by subjecting each fly to a mechanical disturbance by tapping the vial twice against a surface and recording the amount of time the fly is active in the 45 second period immediately following the disturbance. All measurements were taken from 8 a.m.–12 p.m., 2–6 hours after lights on. Startle response scores were obtained for 2 replicates of 15 males and 15 females for each of the 192 lines in a randomized design for both control and MSB exposure conditions. To account for temporal fluctuations and sampling assay bias, a block design was implemented, where 15–20 lines were tested during a two week period for each block.

### Negative geotaxis assay

Single 13–16 day old flies, collected under CO_2_ exposure into vials containing 5 ml of standard cornmeal-agar-molasses media were left overnight to acclimate to the new environment. Flies were transferred to glass assay tubes (Pyrex-Corning flat bottom, rimless culture tubes #9850-25) during 8 a.m.–12 p.m., 2–6 hours after lights on, the next day. To measure negative geotaxis, flies were tapped to the bottom of the glass tube and allowed to climb up along the wall of the tube for a period of 5 seconds. Each fly received a score for the highest point reached during the assay period according to 26 divisions of 5 mm each so that scores ranged from 0 to 26. Geotaxis scores were obtained for 2 replicates of 15 males and 15 females for each of the 192 lines for both control and MSB exposure conditions in the same randomized block design as startle response.

### Quantitative genetics of startle response and negative geotaxis

To test for effects of treatment (control *vs.* MSB supplemented medium), we used the full mixed model ANOVA *y = μ*+*B*+*L*(*B*)+*S*+*T*+*S*×*L*(*B*)+*S*×*T*+*T*×*L*(*B*)+*S*×*T*×*L*(*B*)+*R*(*S*×*T*×*L*(*B*))*+E*. The model was used to partition variation between blocks (*B*, random), line within blocks (*L*(*B*), random), sex (*S*, fixed), treatment (*T*, fixed), and all interactions, vial replicate (*R*, random), and error (*E*). When a significant block term was found, we corrected by subtracting the overall mean by block, treatment, and sex from each corresponding raw data point. To ensure a positive number we then added back the overall mean by sex and treatment: [(*x_i_*_(BST)_−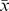
_(BST)_)+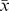
_(ST)_]. Using the transformed data, we removed block effects and tested for the effects of treatment using the mixed model ANOVA *y = μ*+*L*+*S*+*T*+*S*×*L*+*S*×*T*+*T*×*L*+*S*×*T*×*L*+*R*(*S*×*T*×*L*)+*E*. Correlations across environments were calculated as *r_GE_* = *σ* ^2^ *_L_/(σ* ^2^ *_L_*+*σ* ^2^ *_LT_*+*σ* ^2^ *_LST_*). In addition, we performed reduced analyses within each treatment for the block-corrected data using mixed model ANOVAs of form *y = μ*+*L*+*S*+*L*×*S*+*R*(*L*×*S*)+*E*. Broad sense heritabilities were estimated as *H* ^2^ *= *(*σ* ^2^ *_L_*+*σ* ^2^ *_LS_*)*/*(*σ* ^2^ *_L_*+*σ* ^2^ *_LS_*+*σ* ^2^ *_E_*), and genetic correlations between sexes and traits were estimated as *r_G_ = cov*_12_ */*(*σ*_1_×*σ*_2_), where 1 and 2 represent either males and females or control and MSB medium [34].

### Quantitative genetics of sensitivity

In order to identify which lines are more sensitive or resistant to MSB treatment, we computed the measure of sensitivity as [(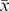
_C *Linei*_−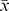
_MSB *Linei*_)/(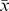
_C *Pop*_−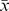
_MSB *Pop*_)], which is the difference in individual line means under control and MSB treated conditions divided by the difference in overall population mean in both control and MSB conditions [35]. The variance of means across the two treatments was estimated as *σ* ^2^ *_M_ = *0.25(*σ* ^2^ *_L C_*+*σ* ^2^ *_L MSB_*)+0.5*σ* ^2^ *_L C, MSB_*. The variance of sensitivity across pairs of environments was estimated as *σ* ^2^ *_S_ = *(*σ* ^2^ *_L C_*+*σ* ^2^ *_L MSB_*−*2σ* ^2^ *_L C, MSB_*)*/D* ^2^. The difference between means is *D* = 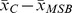. The covariance between the mean and sensitivity was estimated as *cov_MS_ = *(*σ* ^2^ *_L C_−σ* ^2^ *_L MSB_*)/*2D*. The genetic correlation between the mean and sensitivity is calculated as *r_MS_ = cov_MS_*/(*σ_M_*×*σ_S_*) [35].

### Genome-wide association (GWA) study

All analyses were performed on line means of 167 of the 192 DGRP lines [31]. Genotype-phenotype associations were performed on 2,490,165 segregating bivariate single nucleotide polymorphisms (SNPs) that were present in four or more DGRP lines. Marker associations were tested using the ANOVA mixed model *y = μ*+*M*+*S*+*M*×*S*+*L*(*M*×*S*)+*E*, where *M* is the fixed effect of the polymorphic site, *S* is the fixed effect of sex, and *L*(*M*×*S*) is the random effect of line within marker and sex. Reduced analyses were performed on males and females using the fixed effects reduced model *y = μ*+*M*+*E*. An arbitrary threshold of *P*<10^−5^ was used to nominate SNPs for further study. Effects (*a*) of SNPs were estimated as the average difference in trait mean between the major and minor alleles (the major allele is the more frequent allele in the population).

To estimate the fraction of total phenotypic and genetic variation accounted for by markers, we used multiple regression, because single marker analysis can lead to biased estimates of allelic effects when multiple markers jointly affect the trait [31]. Gene-centered forward regression was used to calculate multiple regression models, starting with all nominally SNPs significant (*P*<10^−5^) within 1 kb of an annotated gene. The most significant marker from the GWA was first fit into the model, and subsequent markers were added until the maximum *r^2^* was attained. Missing marker data SNPs in the model were imputed based on nearest marker information for the final model.

### Mutant validation

We tested whether mutations in 16 of the candidate genes in which polymorphisms were associated with sensitivity/resistance to ROS-induced behavioral deficits also affected MSB effects on geotaxis and startle behaviors. The mutations were homozygous *P{MiET1}* elements inserted within candidate genes, in a common co-isogenic *w^1118^* background [36]. Startle response and negative geotaxis were quantified for eight mutants and the corresponding control lines using the same assays described above. Two replicates of 15 males and 15 females aged 14 days were assessed for all lines reared on both control and 3 mM MSB-supplemented medium. We tested for differences among mutant and control genotypes using the fixed effects ANOVA y = *μ*+*G*+*S*+*T*+*G*×*S*+*G*×*T*+*T*×*S*+*G*×*S*×*T*+*E* for sexes pooled and the reduced ANOVA y = *μ*+*G*+*T*+*G*×*T*+*E* for sexes separately, where *G* is the fixed effect of genotype (mutant or control), *S* is the fixed effect of sex, and *T* is the fixed effect for treatment (MSB or control media), and all interactions. A significant *G*×*T* term indicates that the mutant genotype behaves differently in the treatment environments than the control genotype. Within the MSB environment, we performed comparisons of line means using Dunnett's test to nominate significant differences between mutants (*P{MiET1}* elements) and its control (*w^1118^*).

### Bioinformatics and network analysis

Gene ontology analysis of all genes harboring SNPs associated with variation in sensitivity to chronic oxidative stress was performed with DAVID [37]. Likely cellular interactions between their gene products were identified using R Spider (www.bioprofiling.de) [38].

## Results

### Dose-dependent effects of MSB on survival

**Figure 1:**
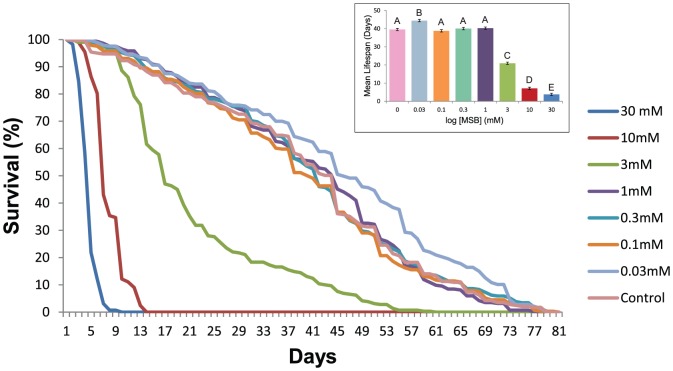
Dose response curve for survival on varying concentrations of Menadione Sodium Bisulfite (MSB). Survival (percent alive) plotted against day for 7 different concentrations of MSB for 5 DGRP lines. Data are averages of all replicates and lines. Inset: Mean lifespan of all lines in days for different concentrations of MSB. Mean lifespan of lines with the same letter are not statistically different (*P*<0.05) from each other.

To determine an optimal discerning MSB concentration to assess natural variation in sensitivity to oxidative stress, we chose five DGRP lines with a range of startle responses under standard conditions [32] and monitored their survival on different concentrations of MSB (Figure 1). Concentrations of MSB above 10 mM caused accelerated death (Figure 1), whereas concentrations below 1 mM did not produce a significant difference from the control. Interestingly, however, the lowest concentration of 0.03 mM MSB increased lifespan by nearly 4 days (Figure 1), consistent with the notion that low levels of ROS can be beneficial for cellular homeostasis [1]. Treatment with 3 mM MSB resulted in death of 50% of flies within 17 days, and was selected as the optimal concentration for further experiments.

### Quantitative genetic analyses of variation in sensitivity to chronic oxidative stress

To assess whether there is genetic variation in sensitivity to chronic oxidative stress in the DGRP, we quantified startle response and negative geotaxis of 13–16 day old flies from 192 DGRP lines reared on standard medium or 3 mM MSB supplemented medium, in a randomized block design (Table S1). We detected a significant block effect (Table S2) for startle response and corrected the phenotypic values for subsequent analysis of startle response to remove this effect.

**Figure 2:**
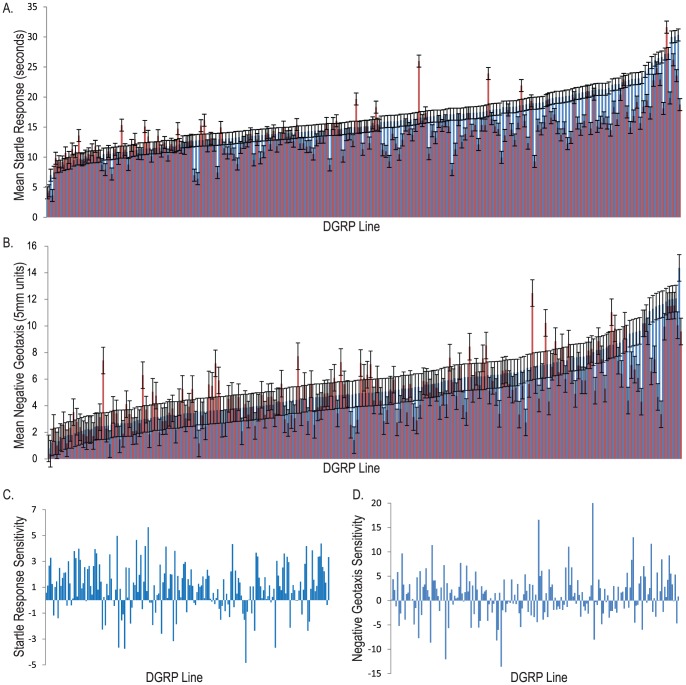
Variation for locomotor behavior in the DGRP. (A) Histogram of line means for startle response and (B) negative geotaxis for 192 DGRP lines (13–16 day old flies). The red bars denote 3 mM MSB supplemented medium, and blue bars the control medium. Line means are ranked from smallest to largest on the control medium. (C) Histogram of mean sensitivity means for startle response and (D) negative geotaxis for 192 DGRP lines. Sensitivity is computed as [(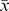
_C *Line i*_−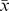
_MSB *Line i*_)/(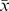
_C *Pop*_−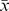
_MSB *Pop*_)].

We found substantial variation in locomotor performance assays among the lines under both control and MSB-treated conditions (Table 1, Figure 2a and b). The significant line by treatment interaction terms for both assays indicates that the lines respond differently to the MSB treatment (*i.e.*, there is genotype by environment interaction for locomotor performance, or equivalently, variation in sensitivity among the lines). This is also evident from the complex pattern of crossing reaction norms (Figure S1), a hallmark of genotype by environment interaction. Thus, we expect to be able to map genetic variants associated with the differential locomotor responses between the control and MSB-induced oxidative stress treatments.

**Table 1:** Analysis of variance of locomotor behavior on control and MSB-supplemented media.

Trait	Source	df	MS	F	*P*	*σ* ^2^
Startle	Line (*L*)	191	1951.11	5.84	<0.0001	13.78
Response*	Sex (*S*)	1	4549.86	48.89	<0.0001	Fixed
	Treatment (*T*)	1	212463	72.06	<0.0001	Fixed
	*L*×*S*	191	93.51	1.59	0.0008	0
	*T*×*S*	1	62.24	1.06	0.3045	Fixed
	*L*×*T*	191	299.66	5.09	<0.0001	3.36
	*L*×*T*×*S*	191	58.92	0.54	1.0000	0
	Replicate(*L*×*T*×*S*)	768	110.34	4.10	<0.0001	4.82
	Error	21,593	26.90			26.90
Negative	Line (*L*)	191	597.79	4.56	<0.0001	4.01
Geotaxis	Sex (*S*)	1	16082	214.40	<0.0001	Fixed
	Treatment (*T*)	1	770.09	8.71	0.0035	Fixed
	*L*×*S*	191	76.77	2.08	<0.0001	0.36
	*T*×*S*	1	21.26	0.58	0.4473	Fixed
	*L*×*T*	191	31.30	2.47	<0.0001	0.97
	*L*×*T*×*S*	191	36.90	0.69	0.9989	0.22
	Replicate(*L*×*T*×*S*)	766	53.46	1.75	<0.0001	0.66
	Error	21,593	26.90			26.90

df: degrees of freedom; MS: Type III Mean Squares; F: F-statistic; *P*: *P*-value; *σ* ^2^: Variance component.

* Raw phenotypic data were corrected to remove the block effect.

Reduced ANOVAs within each environment showed significant variation among lines for both behaviors (Table S3). Broad sense heritabilities for startle response are *H* ^2^ = 0.41 for both control and MSB treated flies, and *H* ^2^ = 0.14 and *H* ^2^ = 0.15 for negative geotaxis for control and MSB treated flies, respectively, when pooled across sexes. The line by sex interaction is significant for startle response for flies reared on MSB medium, but the absolute effect is small, with a cross-sex genetic correlation of *r_G_* = 0.95. Thus, we expect most variants associated with locomotor behavior in the two treatments will be common to males and females, although some fraction will be sex-specific or sex-biased.

Startle response and negative geotaxis both have a startle component, but the geotaxis trait is directional, while startle response is not (necessarily) directional. Not surprisingly, the two traits are positively correlated within each treatment, but the correlations are moderate (*r_G_* = 0.60, *r_P_* = 0.53 for the control treatment; *r_G_* = 0.55, *r_P_* = 0.50 for the MSB treatment) (Figure S2). Therefore, we expect to map variants associated with both traits as well as for each trait separately.

To enable direct comparisons between the magnitude of genotype by environment interaction for startle behavior and negative geotaxis, we quantified interaction using a sensitivity score [35] for each line (Figure 2c, d). There is considerable genetic variation in sensitivity among the 192 lines for both behaviors, reflecting the significant line by treatment interaction term. Although not large, the difference in overall mean for startle response (−1.93 s) and geotaxis (−0.41 units) between the control and MSB treatments was significant (Table 1) and negative, indicating overall decreased performance as a result of chronic MSB exposure. However, the sensitivity scores reveal that the line by treatment interaction is not only due to variation in relative decline in performance when exposed to chronic oxidative stress, but that many lines were unaffected by exposure and others actually had improved performance after MSB exposure (Figure 2). Thus, the DGRP population harbors genetic variants that facilitate resistance to chemically induced oxidative stress. Furthermore, although the mean performance for startle response and negative geotaxis are positively correlated in the two treatments, the sensitivities are poorly correlated (*r_P_* = 0.14) (Figure S3). Therefore, we expect to map different variants associated with genotype by environment interaction for the two locomotor phenotypes since the underlying genetic mechanisms are largely independent.

Finally, we assessed the extent to which sensitivity is correlated with mean locomotor behavior across the two treatments. We calculated the cross-environment genetic correlation (*r_G_*, a measure of the extent to which the means are correlated across environments), and the correlation (*r_MS_*) between mean performance and sensitivity [35] (Table S4). Despite the highly significant contribution of genotype by environment interaction, the genetic correlations between control and MSB exposure for each behavior are high (*r_G_* = 0.82 for both startle response and negative geotaxis, Table S3) (Figure S4). However, the sensitivities are poorly correlated with mean performance (*r_MS_* = 0.29 for startle response and *r_MS_* = 0.10 for negative geotaxis) (Figure S5). Thus, we expect to map largely different variants affecting locomotor performance *per se*, and sensitivity.

### Genome-wide association mapping

**Figure 3:**
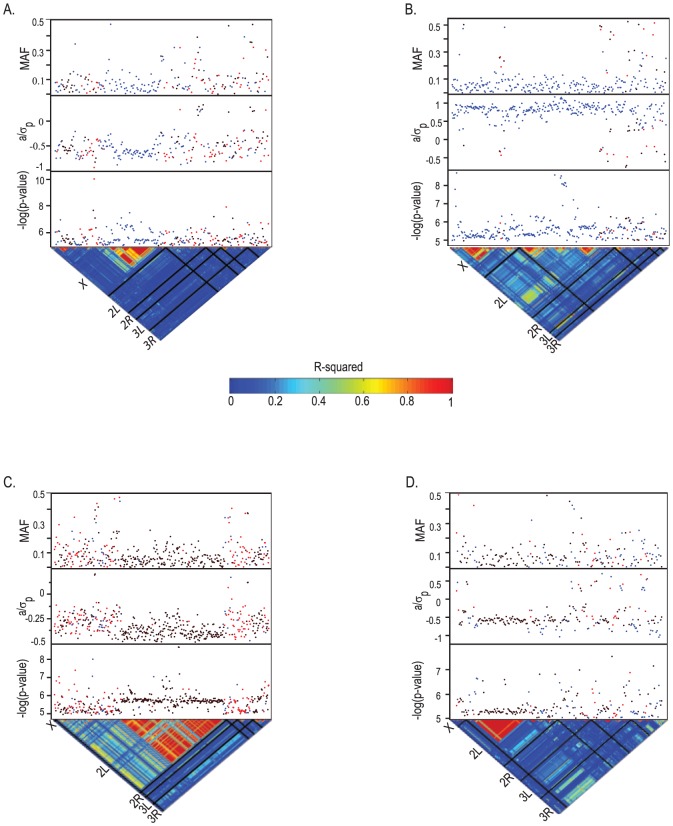
Genome-wide association analyses for locomotor behavior. All SNPs with *P*<10^−5^ are represented by the lowest *P*-value for the average of males and females (black), males (blue) or females (red). The upper panels give the SNP minor allele frequency (MAF), scaled effect size (*a*/*σ_P_*), and −log(*P*-value). The lower panel displays the degree of LD (*r^2^*) between SNPs for the five major chromosome arms, separated by black bars. (A) Startle response on MSB medium. (B) Startle response sensitivity. (C) Negative geotaxis on MSB medium. (D) Negative geotaxis sensitivity.

We performed four genome-wide association analyses using 2,490,165 SNPs present in four or more lines [31] from the full sequence data of 167 DGRP lines: for startle response and negative geotaxis of flies reared on MSB; and for startle response and negative geotaxis sensitivity. At a nominal significance threshold of *P*<10^−5^, we found 251 SNPs in 235 genes associated with startle response in the presence of MSB; 291 SNPs in 244 genes associated with startle response sensitivity; 468 SNPs in 227 genes associated with negative geotaxis in flies treated with MSB; and 220 SNPs in 192 genes associated with negative geotaxis sensitivity (Figure 3, Table S5). At *P*<10^−6^, there are 52 SNPs associated with startle response in the presence of MSB, 41 SNPs associated with startle response sensitivity, 53 SNPs associated with negative geotaxis, and 33 SNPs associated with negative geotaxis sensitivity (Figure 3, Table S5). These significance thresholds correspond to approximate false discovery rates of 10% (*P*<10^−5^) and 6% (*P*<10^−6^). It is not possible to compute true false discovery rates because SNPs are not independent, and thus the correct value for the numerator and denominator are not known. Although linkage disequilibrium (LD) declines rapidly on average with physical distance [31], there is great variation about the average. For example, there is a large LD block of 228 SNPs on chromosome *2L* associated with variation in negative geotaxis following MSB treatment for which the average *R* ^2^>0.8 (Figure 3c).

**Figure 4:**
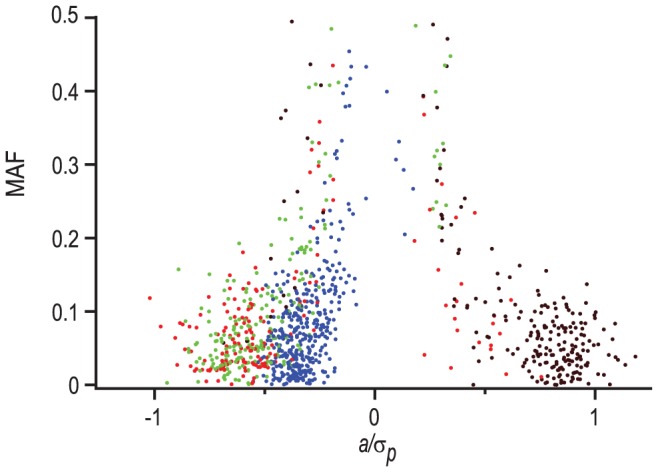
Minor allele frequency and scaled effect size. All SNP effects and corresponding minor allele frequencies are shown for all traits: startle response on MSB medium (green), startle response sensitivity (black), negative geotaxis on MSB medium (blue), and negative geotaxis sensitivity (red).

The minor allele frequency for most of the associated SNPs is <0.15, and there is an inverse relationship between effect sizes and minor allele frequency (Figure 4), as found for other quantitative traits in previous studies [31], [39]. The minor allele class was associated with alleles that both increase and decrease behavioral phenotypes as well as their sensitivities, as would be expected for traits under stabilizing natural selection.

As expected from the quantitative genetic analyses, there was little overlap of SNPs associated with performance of the two traits on MSB and their sensitivity, but 28 genes were in common between performance on MSB and sensitivity for startle response, and 13 genes were in common between performance on MSB and sensitivity for negative geotaxis (Table S6). A total of 4 SNPs and 15 genes were in common between startle response and negative geotaxis for performance on MSB, while no SNPs but 13 genes were in common between sensitivity for startle response and negative geotaxis (Table S6).

We cannot infer the fraction of phenotypic variation accounted for by the genetic associations from the single marker analyses, because estimates of single marker effects are biased by unknown magnitudes when multiple factors jointly contribute to variation in the trait, and the individual SNPs are not independent due to local LD in some genomic regions and spurious long-range LD imposed by the finite sample size [31]. Therefore, we used multiple regression analysis to estimate effects of multiple SNPs simultaneously, and assess the contribution to the total variance. We restricted these models to a maximum of 12 SNPs to avoid over-parameterization, and focused on SNPs within ±1 kb of annotated genes to facilitate biological interpretation. In contrast to human genome wide association studies, we find that models with 8–12 SNPs explain 48–67% of the phenotypic variation (Table S7). Interestingly, the majority of these SNPS are in introns of genes with predicted transcripts of unknown function.

### Functional tests

Next, we sought to confirm whether novel genes in which naturally occurring variants are associated with variation in susceptibility to chronic oxidative stress show similar associations for mutant alleles. We chose candidate genes for these tests based on the availability of co-isogenic homozygous *P{MiET1}* mutant alleles, GWA significance level, and expression in the brain. Based on these criteria, we selected 16 candidate genes with homozygous *P{MiET1}* element insertions in a common co-isogenic *w^1118^* background, and assessed the effects of MSB exposure on startle behavior and negative geotaxis compared to untreated controls. These included genes associated with axon guidance (*Lar*, *NetA*, and *side*), regulation of transcription (*A2bp1*, *Vsx2*, *luna*, *CG33291*), calcium and calmodulin binding and transport (*igl*, *CG42430*, *Eip63-1*), receptor signaling (*form3*, *CG34411*, *CG13579*), actin organization (*spir*), and regulation of apoptosis (*DLP*). We measured startle responses for *NetA*, *CG34411* (2 insertions), *Lar, DLP*, *spir*, *A2bp1*, *beatIV*, *Vsx2* mutants, and negative geotaxis for *form3*, *Lar*, *Eip63-1*, *luna*, *CG13579*, *side*, *igl*, and *CG33291* mutants.

**Figure 5:**
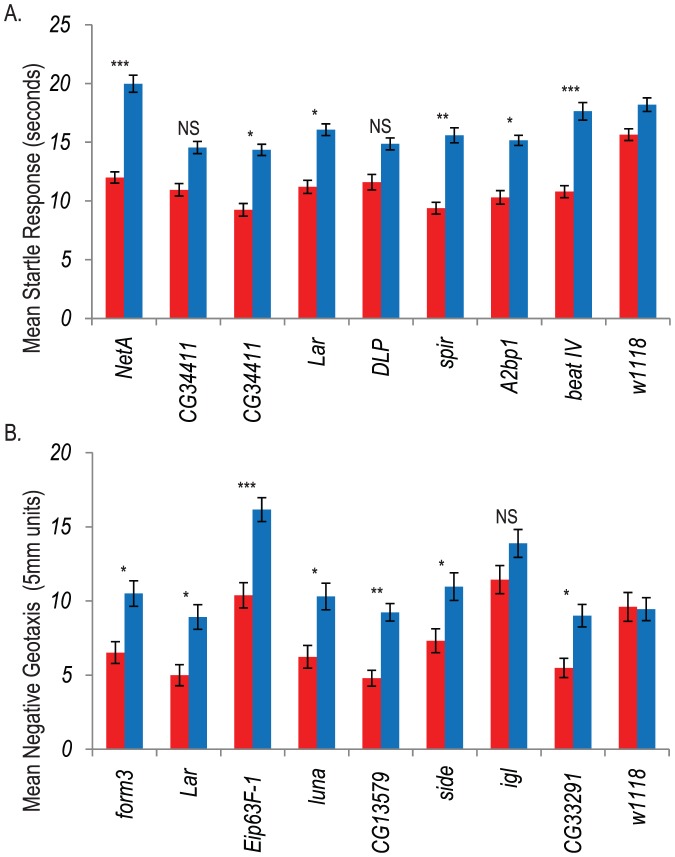
Effects of mutations in candidate genes affecting locomotor behavior under chronic oxidative stress. Mean startle responses (sexes pooled) of homozygous *P{MiET1}* mutations and the co-isogenic control (*w^1118^*) on control medium (blue bars) and MSB supplemented medium (red bars). (A) Startle responses for *NetA^22841^*, *CG34411^23615^*, *CG34411^23395^*, *Lar^24058^*, *DLP^24081^*, *spir^24237^, A2bp1^24263^*and *beatIV^24710^*. (B) Negative geotaxis for *form3^23411^*, *Lar^24058^*, *Eip63-1^24716^*, *luna^25222^*, *CG13579^25600^*, *side^25649^*, *igl^27748^* and *CG33291^27757^*. Significance is from the genotype by treatment term from ANOVA *: *P*<0.05; **: 0.05<*P*<0.01, ***: 0.01<*P*<0.0001; ns: P<0.05.

We used significant genotype by treatment terms in ANOVAs as the measure of sensitivity to MSB, since this term indicates that the mutant phenotype changed as a result of MSB treatment and was not due to an overall locomotor deficit caused by the *P{MiET1}* element insertion. Six of eight mutants tested for altered sensitivity of startle behavior to MSB treatment and seven of eight mutants tested for altered sensitivity of negative geotaxis to MSB treatment showed a significant genotype by treatment effect (Figure 5). This high confirmation rate is consistent with the approximate false discovery rates from the association analyses, and indicates that the genes implicated by these analyses are enriched for true causal associations. This provides a favorable scenario for further bioinformatics analysis to extract mechanistic information from the ensemble of SNPs associated with variation in MSB-induced oxidative stress susceptibility.

### Identification of a neural cellular network for susceptibility to chronic oxidative stress

To evaluate whether genes implicated in sensitivity/resistance to age-related decline in behavioral performance from MSB-induced oxidative stress are functionally related, we first performed a gene ontology enrichment analysis (Table S8). This analysis revealed that the entire suite of genes associated with oxidative stress is enriched for processes associated with neuronal development. In addition, genes associated with neuronal function, including ion channel and transmembrane transport activities, were significantly over-represented. Furthermore, protein domain analysis displayed an over-representation of immunoglobulin-like genes. These analyses suggest that the candidate genes with SNPs associated with chronic oxidative stress susceptibility include an over-representation of neural development, immunity, and signaling genes, indicating a link between sensitivity to oxidative stress and neural function.

**Figure 6:**
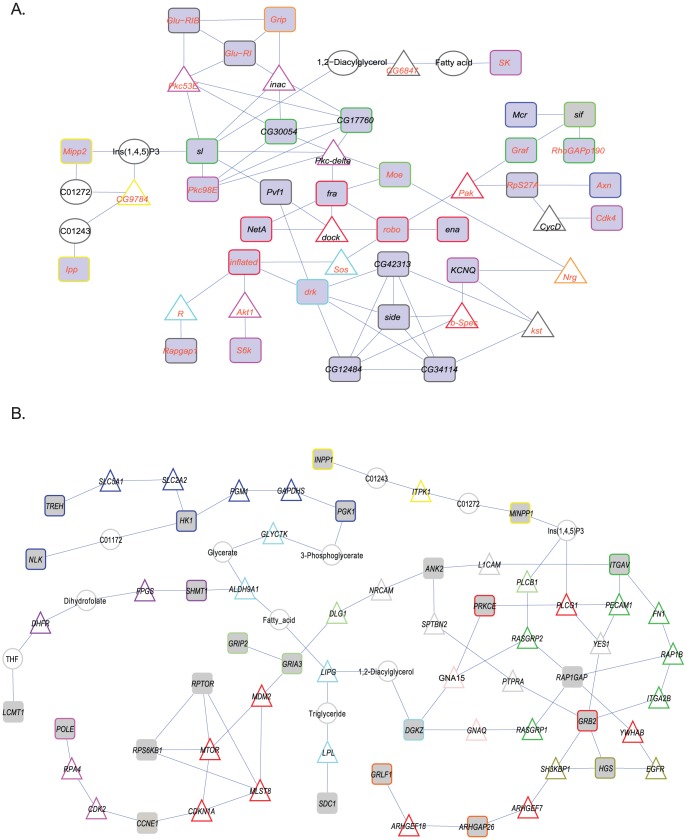
Cellular networks of candidate genes. (A) Enriched cellular genetic pathway for candidate genes from all genome wide association analyses (gray squares), allowing one missing gene (white triangles) or compound (white circles). The border colors indicate the over-represented gene ontology categories (*P*<0.005): axon guidance (red), synapse organization (orange), protein phosphorylation (magenta), signal transduction (dark green), inositol phosphate metabolism (yellow), phagocytosis engulfment (dark blue), regulation of cell shape (light blue), actin cytoskeleton organization (light green), and potassium ion transport (pink). Drosophila genes with human homologs are indicated in red font. (B) Enriched network for human homologs of Drosophila candidate genes (gray squares) missing no more than two consecutive genes (white triangles) or compounds (white circles). The border colors indicate the over-represented gene ontology categories (*P* = 0.085): Rho GTPase signaling (orange), NGF signaling (red), EGFR signaling (olive green), synaptic transmission (light green), integrin cell surface interactions (dark green), DNA replication (magenta), inositol phosphate metabolism (yellow), integration of energy metabolism (light pink), metabolism of vitamins and cofactors (dark blue), glycerolipid metabolism (light blue), and metabolism of carbohydrates (light purple).

Next, we performed a more detailed analysis that enabled us to place a subset of these genes in an interconnected network. To accomplish this we used the R spider program [38], which organizes gene products into cellular pathways based on the Reactome signaling network and the KEGG metabolic network to determine if interactions are over-represented more than expected by chance. Using a model that allows for no more than one missing gene or compound between our candidate genes, we found a significantly enriched network (*P*<0.005), comprising 32 candidate genes for which natural variation is associated with variation in oxidative stress-induced behavioral decline (Figure 6a). The network that emerged from this analysis revealed that genes that harbor alternative alleles associated with susceptibility/resistance to chronic oxidative stress are functionally connected through processes that encompass axon guidance and synapse organization, ion transport, glutamate receptor signaling, inositol triphosphate signaling and protein phosphorylation (Figure 6a).

Among the candidate genes in our screen, 205 have human homologs (Table S9). We performed the same analysis using the human homologs, except that here we allowed no more than two missing genes between homologs. This analysis again revealed a network of gene products associated with inositol triphosphate signaling and synaptic transmission and, in addition, implicated ensembles of gene products associated with intermediary metabolism, signaling by NGF, EGFR, and Rho GTPases, and DNA replication (*P* = 0.085, Figure 6b). Our results show that individual variation in susceptibility to the effects of chronic oxidative stress on behavior may at least in part be determined by polymorphisms that affect subtle variation in neural connectivity and function.

**Figure 7:**
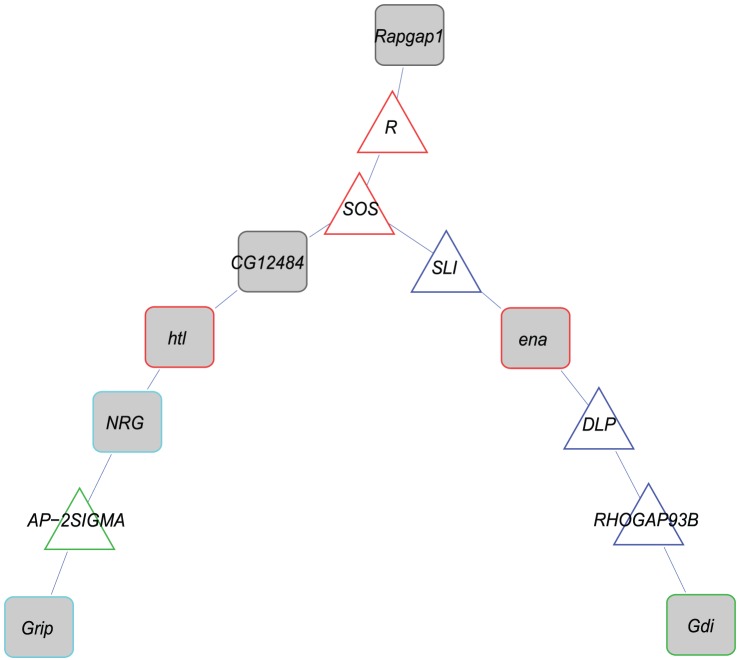
Cellular networks for common candidate genes between acute and chronic oxidative stress. The network depicts the candidate genes (grey squares) with no more than two missing genes. Border colors depict the enriched (*P*<0.005) gene ontology categories of synapse organization (light blue), axon guidance (dark blue), vesicle mediated transport (green) and regulation of cell shape (red).

A recent genome-wide association study on the DGRP which measured accelerated death induced by acute oxidative stress induced with high doses of MSB and paraquat [39] identified 76 genes that are in common with this study (Table S10). These 76 genes showed enrichment for immunoglobulin and immunoglobulin-like genes (Benjamini-corrected *P*-value = 3.41×10^−3^; Table S11) and an enriched (*P*<0.005, Figure 7) network associated with the same terms, suggesting that polymorphisms in immune defense genes are associated with sensitivity to both chronic and acute oxidative stress.

## Discussion

We have taken advantage of natural variation in the sequenced, inbred lines comprising the *Drosophila melanogaster* Genetics Reference Panel to identify cellular networks associated with sensitivity/resistance to the effects of MSB-induced chronic oxidative stress on age-related impairment in locomotor phenotypes. The DGRP lines are an excellent resource for assessing the magnitude of genotype by environment variation and its genetic basis, since many genetically identical individuals from each line can be assessed in multiple environments. We found that treatment of adult flies with a concentration of MSB that shortens lifespan by approximately 50% results in a significant, but small, decline in performance in both geotaxis and startle behavior averaged over all DGRP lines. However, there was great variation in the magnitude of the difference between the control and MSB-treated flies (*i.e.*, genotype by environment interaction), whereby some lines were highly sensitive and others actually had improved performance when reared in MSB-supplemented medium. The correlation between sensitivity and the mean performance for each trait was low, indicating that the genotype by environment interaction is not caused by a scale effect [35], and that different variants will affect mean locomotor performance than the difference in locomotion between the two treatments. Startle response and negative geotaxis are significantly genetically correlated within each treatment, but their sensitivities are poorly correlated. Thus, the effect of MSB on locomotion is specific for the different measures of locomotor performance.

Our GWA identified 1,218 SNPs and 796 genes associated with the effects of chronic MSB exposure on geotaxis and startle behavior at a nominal *P*-value<10^−5^. In contrast to results from human GWA studies [40], the effects of variants detected by single marker analysis are not small, and are primarily due to alleles at the low end of the frequency spectrum. The effect sizes are negatively correlated with allele frequency, such that rare alleles have larger effects than common alleles, consistent with previous GWA analyses in this population [31], [39]. Models simultaneously fitting up to 12 SNPs provide better estimates of individual SNP effects and explain from 48–67% of the phenotypic variation for sensitivity. If the genetic architecture of human complex traits is similarly dominated by larger effects of low frequency variants, the missing heritability in human GWA studies [40] may be attributable to underestimation of the effects of the causal SNPs by the common SNPs used in the genotyping assays.

However, testing the effects of over 2.5 million SNPs in only 200 lines presents a statistical problem: there are many different multiple regression models utilizing subsets of SNPs that provide equally good prediction models in terms of variance explained. However, we can utilize the evolutionary conservation of biological pathways and the power of the Drosophila model system to assess which of the variants nominated by the GWA study are potentially true positives and which may be false positives. We hypothesized that the variants identified in the GWA analysis are enriched for true positives, and that these loci are likely to interact in known pathways. Indeed, our bioinformatics analysis reveals an over-representation of candidate genes associated with nervous system development and neural signaling, as well as immune defense, consistent with the established association of exposure to oxidative stress and increased risk for development of neurodegenerative diseases, such as Parkinson's disease, Alzheimer's disease, and amyotrophic lateral sclerosis [4]. We took advantage of a recent collection of *Minos* insertional mutations that were generated in a common isogenic background [36] to query whether these mutations in candidate genes also affected sensitivity of the measured behaviors following exposure to MSB. The validation rate for these tests was 80%, consistent with enrichment of our candidate gene list for true positive associations. In the future, the mechanistic basis of these associations can be probed in Drosophila by taking advantage of the ability to knock down gene expression by RNAi as well as overexpress genes, either ubiquitously or by temporal and spatial control of gene expression.

Approximately 25% of the confirmed genes with mutational effects on locomotion-related behaviors under MSB-induced chronic oxidative stress are evolutionarily conserved and have human homologs. The human homolog of *CG5703*, *NDUFV2*, has been linked to Parkinson's disease [41]. *GRIP2*, the human homolog of *Grip*, has been implicated in Alzheimer's disease [42]. *ANKS1B*, the homolog of *CG4393*, interacts with amyloid beta protein precursor, which has been implicated in the pathogenesis of Alzheimer's disease [43]. *PTPRD*, the human counterpart of *Lar*, interacts with *IL1RAPL1*, which is implicated in mental retardation and autism [44]. The human homolog of *CG7394*, *DNAJC19*, is associated with cardiomyopathy [45]. A large percentage of the human homologs of the other implicated candidate genes have tumorigenic functions, tumor suppressor properties, or other links to human diseases. These results show that studies in the powerful *Drosophila melanogaster* genetic model system can guide future translational research on human diseases associated with exposure to oxidative stress.

Most of the candidate genes implicated by our GWA study are novel and have not previously been associated with either geotaxis or startle behavior or sensitivity/resistance to oxidative stress. This highlights the value of interrogating the effects of natural variants that have survived the sieve of natural selection to understand the genetic architecture of quantitative traits. In this regard, GWA analyses using the DGRP are complementary to traditional mutant screens. On the other hand, we did not detect variants in *Sod* and *Cat*, as well as other loci known to affect sensitivity to oxidative stress, in our analysis. Possibly these loci are under such strong purifying natural selection that they are either invariant or that the variation is too rare to be included in our GWA study, which is blind to variants detected in fewer than four DGRP lines. In addition, we only assessed the effects of SNP variants. In the future, we will be able to evaluate the effects of the full spectrum of naturally occurring mutations, including insertion/deletion mutations, microsatellites and other structural variants. Finally, future systems genetics analyses [46] including the effects of variants segregating in the DGRP on gene expression and other molecular phenotypes will enable us to derive causal molecular networks affecting sensitivity of locomotor phenotypes to oxidative stress.

## Supporting Information

Figure S1 **Reaction norms for locomotor performance on control (C) and MSB-supplemented (T) medium. (A) Startle response. (B) Negative geotaxis.**
(PDF)

Figure S2 **Correlation between startle response and negative geotaxis. (A) Control medium. (B) MSB-supplemented medium.**
(PDF)

Figure S3 **Correlation between sensitivity of startle response and negative geotaxis.**
(PDF)

Figure S4 **Correlation of locomotor behavior between control and MSB-supplemented medium. (A) Startle response. (B) Negative geotaxis.**
(PDF)

Figure S5 **Correlation between mean and sensitivity of locomotor behavior. (A) Startle response. (B) Negative geotaxis.**
(PDF)

Table S1 **Mean startle responses and negative geotaxis scores for DGRP lines.**
(DOCX)

Table S2 **ANOVA of locomotor traits across treatments.**
(DOCX)

Table S3 **ANOVA of locomotor traits within treatments.**
(DOCX)

Table S4 **Quantitative genetic analysis of sensitivity.**
(DOCX)

Table S5 **Significant GWA results.**
(XLSX)

Table S6 **Overlap between SNPs and genes from GWA analysis.**
(DOCX)

Table S7 **Gene-centered prediction models.**
(DOCX)

Table S8 **Gene Ontology enrichment analysis.**
(XLSX)

Table S9 **Human orthologs of Drosophila genes.**
(XLSX)

Table S10 **Overlap of genes in this study and GWA for acute oxidative stress.**
(DOCX)

Table S11 **Gene Ontology enrichment analysis of genes in common between chronic and acute oxidative stress.**
(XLSX)
