# Supplementary figures and images for: Genome-Wide Association for Sensitivity to Chronic Oxidative Stress in Drosophila melanogaster
Source: PLoS One. 2012 Jun 8;7(6):e38722. doi: 10.1371/journal.pone.0038722 (PMC3371005; doi:10.1371/journal.pone.0038722)

A.

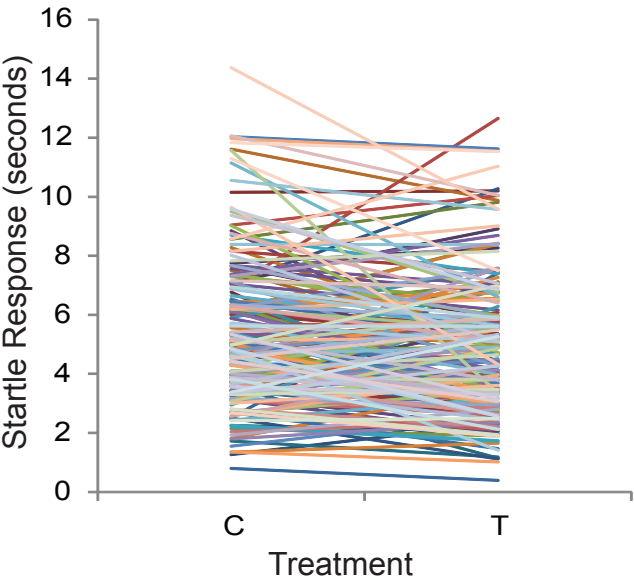

B.

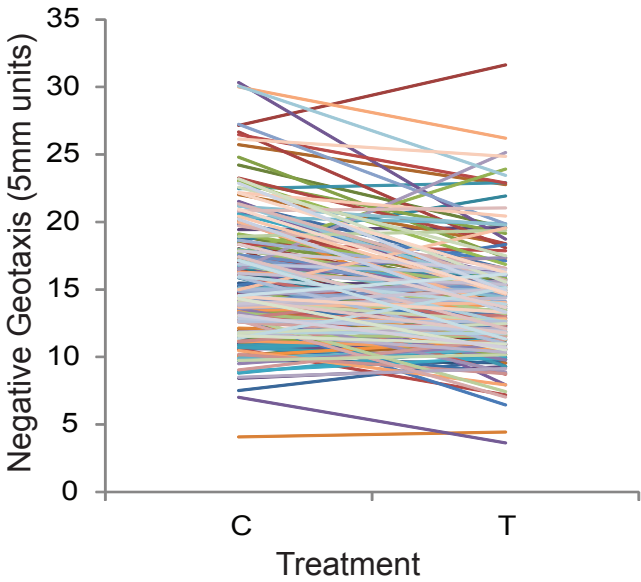

Supplement: Figure S1 — Reaction norms for locomotor performance on control (C) and MSB-supplemented (T) medium. (A) Startle response. (B) Negative geotaxis. (PDF) [file pone.0038722.s001.pdf]

A.

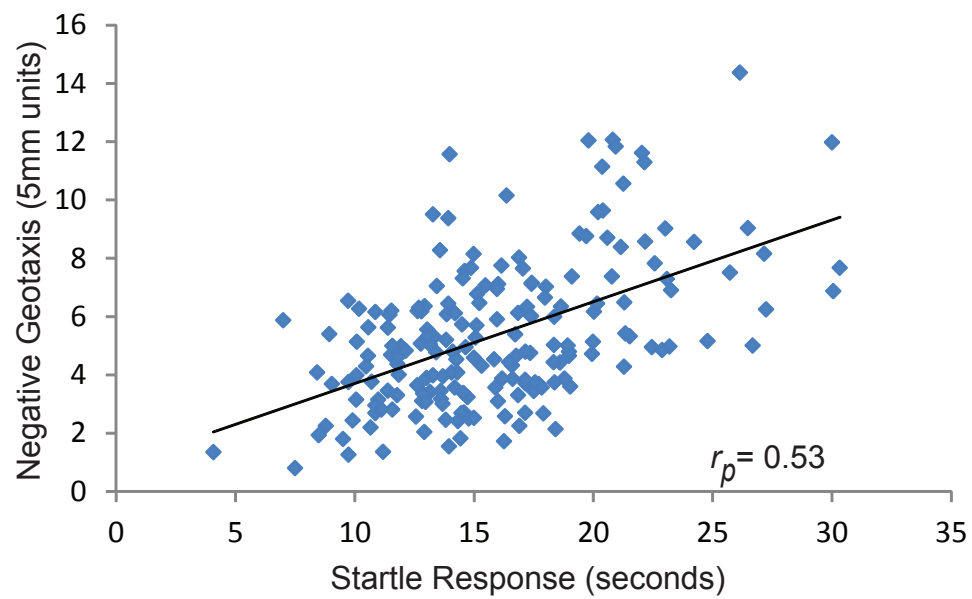

B.

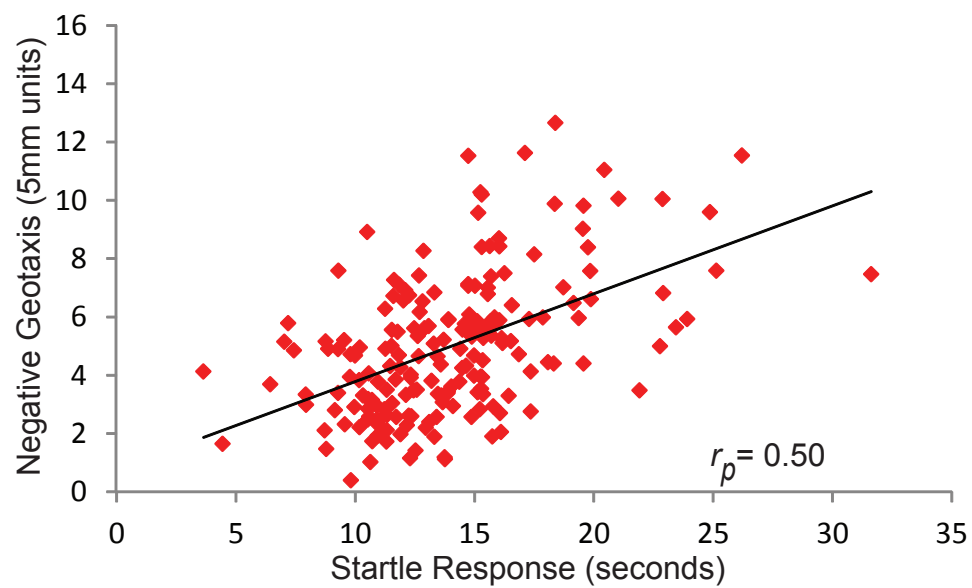

Supplement: Figure S2 — Correlation between startle response and negative geotaxis. (A) Control medium. (B) MSB-supplemented medium. (PDF) [file pone.0038722.s002.pdf]

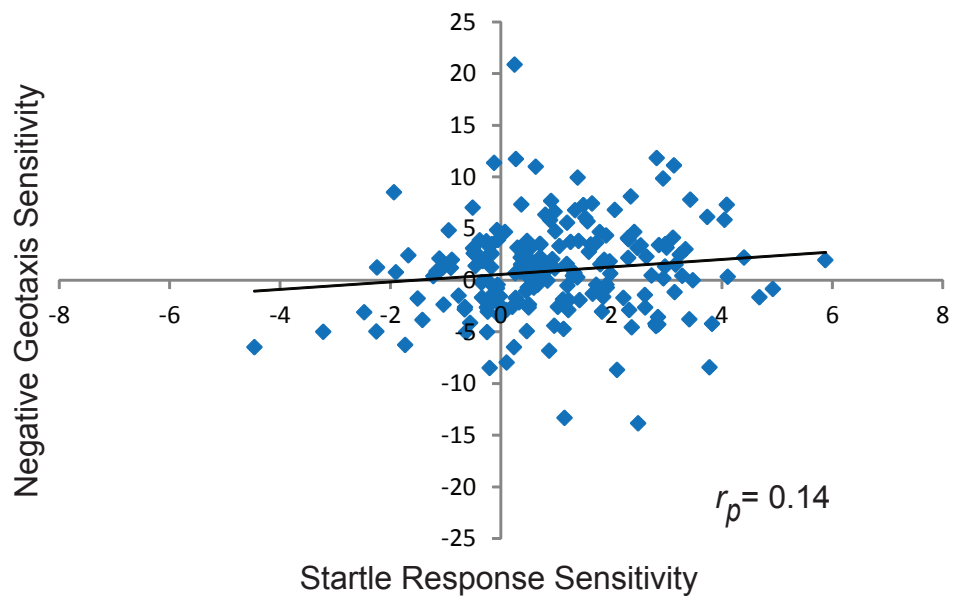

Supplement: Figure S3 — Correlation between sensitivity of startle response and negative geotaxis. (PDF) [file pone.0038722.s003.pdf]

A.

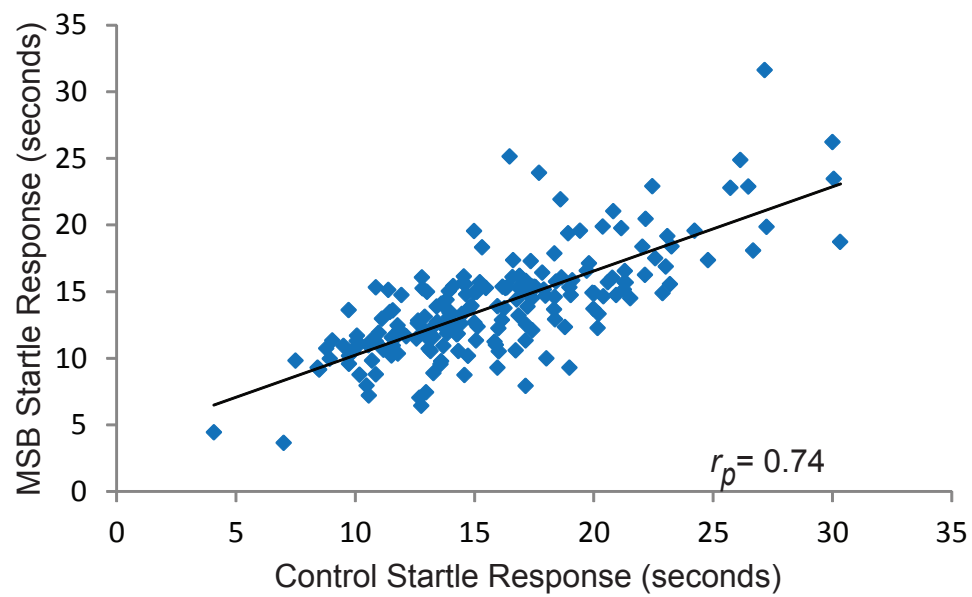

B.

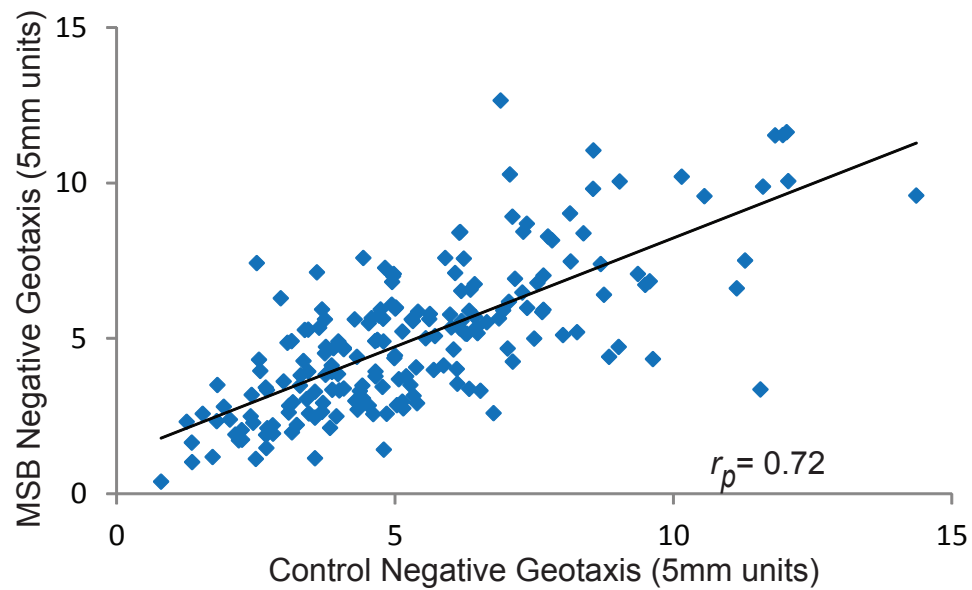

Supplement: Figure S4 — Correlation of locomotor behavior between control and MSB-supplemented medium. (A) Startle response. (B) Negative geotaxis. (PDF) [file pone.0038722.s004.pdf]

A.

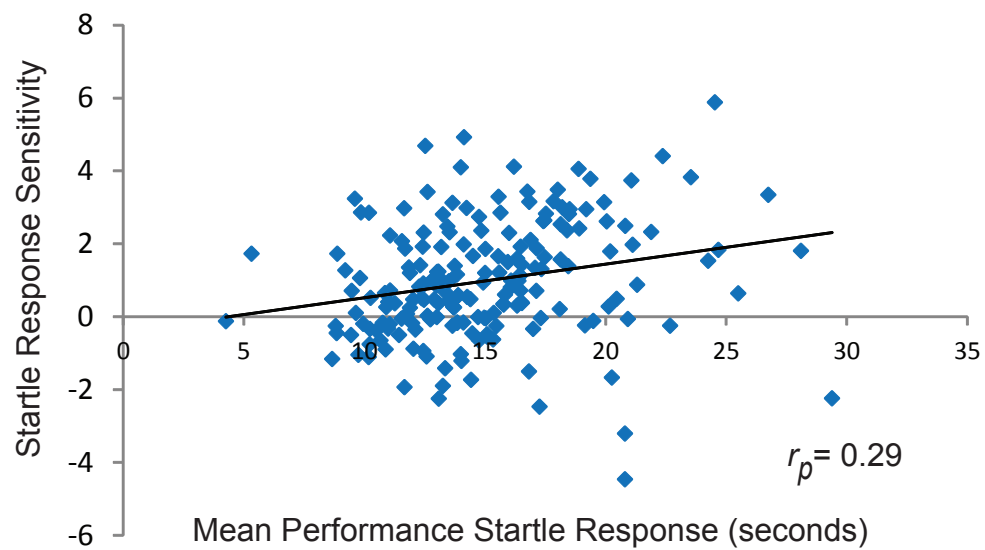

B.

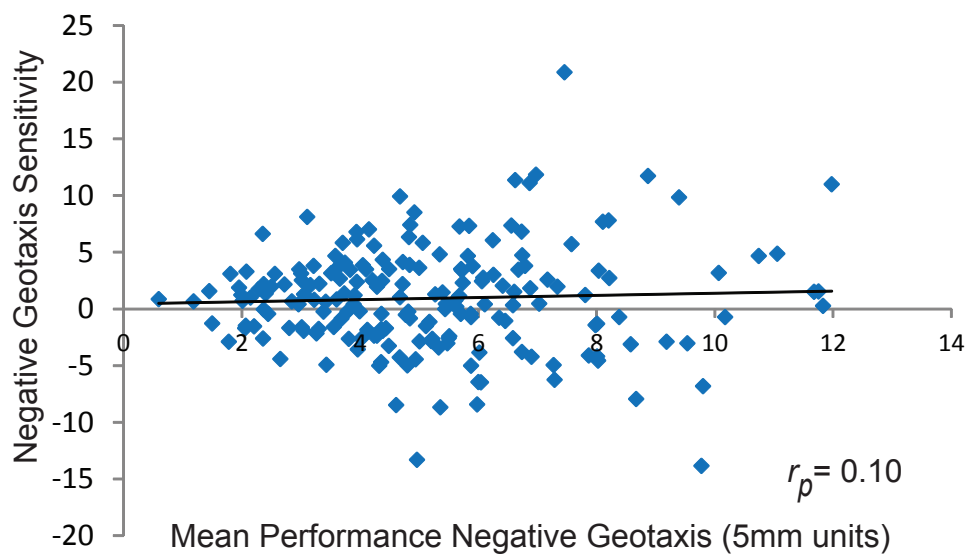

Supplement: Figure S5 — Correlation between mean and sensitivity of locomotor behavior. (A) Startle response. (B) Negative geotaxis. (PDF) [file pone.0038722.s005.pdf]
