# Supplementary material for: Genome-Wide Association for Sensitivity to Chronic Oxidative Stress in Drosophila melanogaster
Source: PLoS One. 2012 Jun 8;7(6):e38722. doi: 10.1371/journal.pone.0038722 (PMC3371005; doi:10.1371/journal.pone.0038722)
Supplement: Table S1 — Mean startle responses and negative geotaxis scores for DGRP lines. (DOCX) [file pone.0038722.s006.docx]

**Supplementary Table 1**

**Mean phenotype values for quantitative traits**

| **Line** | **SR***_♀_**_C_** | **SR***_♂_**_C_** | **SR***_♀_**_MSB_** | **SR***_♂_**_MSB_** | **NG**_♀_**_C_** | **NG**_♂_**_C_** | **NG**_♀_**_MSB_** | **NG**_♂_**_MSB_** |
| --- | --- | --- | --- | --- | --- | --- | --- | --- |
| RAL_21 | 16.76 | 17.04 | 17.83 | 14.39 | 1.97 | 2.53 | 1.04 | 3.06 |
| RAL_26 | 17.38 | 16.72 | 14.14 | 16.10 | 4.97 | 10.33 | 4.10 | 7.59 |
| RAL_28 | 19.97 | 20.10 | 13.69 | 16.10 | 5.57 | 6.77 | 4.13 | 6.50 |
| RAL_38 | 15.07 | 17.22 | 11.66 | 14.06 | 5.61 | 9.90 | 5.89 | 10.64 |
| RAL_40 | 12.80 | 15.59 | 12.24 | 12.36 | 3.13 | 4.00 | 0.82 | 1.47 |
| RAL_41 | 11.74 | 12.16 | 16.67 | 12.83 | 4.82 | 5.13 | 4.03 | 10.10 |
| RAL_42 | 9.69 | 9.79 | 9.78 | 9.35 | 0.70 | 1.82 | 1.75 | 2.87 |
| RAL_45 | 14.37 | 15.88 | 11.52 | 13.20 | 3.33 | 10.21 | 1.27 | 3.91 |
| RAL_49 | 10.31 | 12.59 | 15.06 | 11.86 | 5.29 | 6.83 | 2.21 | 7.07 |
| RAL_57 | 15.68 | 16.35 | 10.29 | 10.72 | 5.19 | 9.03 | 6.07 | 11.75 |
| RAL_59 | 17.47 | 15.05 | 16.38 | 11.12 | 1.71 | 1.73 | 1.19 | 1.18 |
| RAL_69 | 17.93 | 20.04 | 16.21 | 14.43 | 4.93 | 4.37 | 5.32 | 2.54 |
| RAL_73 | 14.40 | 15.16 | 14.96 | 12.56 | 1.33 | 3.67 | 1.45 | 0.79 |
| RAL_75 | 15.27 | 17.46 | 16.20 | 14.41 | 9.11 | 11.20 | 9.86 | 10.53 |
| RAL_83 | 9.64 | 10.51 | 9.97 | 13.42 | 4.16 | 3.79 | 3.19 | 4.50 |
| RAL_85 | 15.58 | 21.19 | 10.91 | 14.90 | 2.04 | 5.45 | 2.72 | 8.48 |
| RAL_88 | 13.70 | 14.90 | 11.83 | 11.83 | 3.17 | 5.00 | 4.34 | 5.03 |
| RAL_91 | 9.04 | 11.15 | 10.29 | 11.50 | 2.67 | 7.60 | 3.40 | 2.53 |
| RAL_93 | 13.44 | 17.52 | 13.32 | 17.20 | 6.03 | 8.10 | 7.65 | 12.90 |
| RAL_101 | 16.08 | 19.96 | 10.56 | 9.42 | 6.57 | 7.48 | 2.41 | 6.92 |
| RAL_105 | 26.59 | 21.86 | 22.29 | 16.85 | 10.33 | 6.79 | 10.17 | 9.45 |
| RAL_109 | 16.34 | 14.12 | 14.94 | 16.48 | 5.00 | 7.93 | 2.47 | 8.23 |
| RAL_129 | 16.50 | 16.96 | 9.69 | 11.46 | 5.81 | 4.96 | 2.81 | 5.32 |
| RAL_136 | 24.47 | 26.99 | 23.73 | 21.83 | 4.04 | 10.97 | 2.30 | 7.68 |
| RAL_138 | 12.32 | 10.47 | 10.32 | 10.87 | 3.71 | 3.20 | 2.52 | 2.65 |
| RAL_142 | 16.20 | 18.59 | 14.17 | 16.60 | 3.48 | 8.55 | 3.40 | 7.30 |
| RAL_149 | 19.54 | 18.52 | 14.06 | 15.42 | 0.90 | 6.30 | 3.31 | 10.93 |
| RAL_153 | 18.67 | 20.18 | 18.22 | 20.93 | 7.00 | 10.70 | 3.67 | 5.14 |
| RAL_158 | 15.84 | 15.85 | 10.25 | 12.20 | 4.20 | 4.87 | 3.57 | 2.10 |
| RAL_161 | 22.95 | 21.13 | 20.32 | 16.40 | 9.90 | 13.33 | 8.52 | 11.23 |
| RAL_176 | 17.73 | 16.59 | 16.69 | 14.89 | 3.00 | 4.41 | 2.52 | 3.33 |
| RAL_177 | 12.77 | 13.54 | 10.67 | 12.45 | 3.50 | 3.33 | 3.55 | 2.53 |
| RAL_181 | 12.60 | 15.12 | 11.76 | 11.93 | 3.67 | 8.50 | 4.63 | 9.56 |
| RAL_189 | 14.44 | 15.02 | 10.21 | 10.16 | 1.11 | 5.37 | 2.39 | 2.03 |
| RAL_195 | 17.40 | 20.19 | 11.44 | 13.23 | 2.80 | 4.94 | 2.00 | 5.83 |
| RAL_208 | 11.63 | 15.52 | 8.81 | 10.28 | 5.45 | 11.10 | 3.46 | 6.93 |
| RAL_217 | 13.92 | 16.09 | 11.88 | 13.47 | 1.00 | 4.04 | 7.61 | 7.23 |
| RAL_223 | 12.97 | 14.42 | 9.92 | 11.93 | 3.17 | 4.73 | 1.60 | 3.37 |
| RAL_227 | 11.65 | 13.95 | 10.19 | 21.91 | 4.04 | 8.31 | 6.67 | 10.16 |
| RAL_228 | 8.75 | 8.09 | 10.01 | 8.57 | 4.44 | 3.72 | 4.27 | 2.50 |
| RAL_229 | 17.21 | 20.02 | 21.31 | 22.52 | 3.21 | 5.62 | 2.82 | 4.13 |
| RAL_233 | 11.97 | 14.69 | 11.76 | 13.19 | 5.21 | 5.40 | 3.60 | 7.61 |
| RAL_235 | 6.79 | 8.22 | 10.01 | 9.63 | 0.67 | 0.93 | 0.62 | 0.17 |
| RAL_237 | 19.10 | 17.62 | 15.08 | 14.13 | 4.73 | 7.25 | 5.46 | 6.07 |
| RAL_239 | 19.40 | 17.42 | 17.24 | 14.26 | 0.97 | 3.30 | 0.77 | 3.04 |
| RAL_256 | 11.45 | 10.54 | 12.27 | 11.53 | 3.00 | 3.30 | 1.35 | 2.59 |
| RAL_272 | 8.24 | 11.22 | 13.70 | 13.53 | 4.77 | 8.31 | 1.41 | 5.20 |
| RAL_280 | 11.72 | 14.35 | 11.38 | 11.66 | 3.07 | 8.03 | 2.90 | 7.10 |
| RAL_287 | 19.74 | 18.22 | 9.58 | 9.00 | 3.17 | 6.42 | 4.21 | 5.59 |
| RAL_301 | 25.13 | 29.19 | 31.62 | 31.63 | 5.78 | 10.54 | 5.90 | 9.03 |
| RAL_303 | 17.50 | 16.95 | 13.38 | 14.37 | 5.83 | 6.83 | 2.89 | 3.86 |
| RAL_304 | 10.43 | 10.69 | 11.35 | 10.57 | 4.17 | 5.14 | 2.69 | 4.86 |
| RAL_306 | 15.53 | 17.65 | 16.89 | 15.27 | 2.31 | 6.34 | 2.72 | 2.68 |
| RAL_307 | 9.56 | 12.19 | 14.60 | 16.02 | 5.69 | 6.62 | 8.00 | 8.79 |
| RAL_309 | 20.94 | 21.66 | 15.99 | 17.07 | 7.07 | 5.90 | 3.89 | 6.45 |
| RAL_310 | 14.21 | 15.10 | 13.87 | 15.69 | 5.31 | 4.58 | 5.41 | 6.74 |
| RAL_313 | 24.16 | 25.42 | 17.19 | 17.53 | 6.10 | 4.21 | 3.93 | 1.57 |
| RAL_315 | 14.70 | 14.52 | 18.22 | 12.90 | 6.32 | 8.80 | 4.18 | 9.38 |
| RAL_317 | 22.85 | 22.08 | 23.17 | 22.64 | 5.56 | 4.33 | 7.52 | 6.11 |
| RAL_318 | 12.67 | 16.19 | 8.84 | 16.34 | 1.38 | 2.25 | 2.86 | 4.13 |
| RAL_320 | 10.65 | 11.08 | 8.55 | 9.03 | 1.93 | 3.45 | 1.10 | 1.83 |
| RAL_321 | 26.31 | 27.05 | 16.51 | 19.65 | 3.73 | 6.28 | 2.34 | 6.54 |
| RAL_324 | 21.23 | 24.95 | 16.62 | 21.70 | 7.79 | 6.79 | 5.85 | 7.10 |
| RAL_325 | 14.06 | 12.82 | 12.20 | 13.23 | 6.27 | 7.83 | 6.71 | 5.62 |
| RAL_332 | 14.88 | 17.04 | 9.10 | 9.48 | 5.17 | 6.63 | 7.00 | 8.16 |
| RAL_335 | 4.56 | 3.59 | 4.91 | 3.98 | 1.07 | 1.63 | 1.76 | 1.54 |
| RAL_336 | 13.12 | 14.50 | 12.18 | 12.16 | 1.52 | 3.40 | 2.53 | 2.03 |
| RAL_338 | 19.34 | 20.56 | 14.91 | 14.89 | 5.17 | 4.27 | 6.07 | 5.39 |
| RAL_340 | 10.84 | 12.22 | 8.79 | 11.60 | 2.30 | 7.07 | 2.86 | 7.03 |
| RAL_350 | 30.15 | 30.53 | 17.90 | 19.56 | 8.79 | 6.55 | 7.03 | 7.00 |
| RAL_352 | 10.57 | 11.15 | 11.72 | 10.81 | 3.00 | 2.90 | 5.96 | 6.60 |
| RAL_356 | 11.29 | 12.98 | 12.44 | 10.83 | 4.17 | 5.48 | 6.70 | 7.83 |
| RAL_357 | 11.70 | 11.45 | 13.18 | 14.01 | 3.70 | 6.28 | 3.93 | 4.80 |
| RAL_358 | 22.83 | 23.69 | 17.95 | 18.83 | 7.57 | 6.23 | 10.70 | 14.60 |
| RAL_359 | 13.62 | 12.40 | 13.52 | 16.45 | 1.87 | 5.93 | 2.83 | 6.52 |
| RAL_360 | 6.70 | 7.32 | 3.95 | 3.33 | 4.71 | 7.03 | 4.76 | 3.50 |
| RAL_361 | 9.68 | 11.74 | 9.66 | 9.97 | 2.52 | 5.00 | 3.53 | 5.90 |
| RAL_362 | 20.66 | 19.65 | 12.56 | 11.97 | 6.46 | 6.41 | 5.17 | 8.31 |
| RAL_365 | 15.17 | 15.46 | 19.52 | 17.14 | 1.63 | 7.00 | 3.93 | 4.87 |
| RAL_367 | 9.98 | 11.16 | 6.87 | 7.52 | 4.33 | 6.93 | 4.83 | 6.73 |
| RAL_370 | 14.35 | 15.77 | 12.24 | 10.42 | 4.55 | 6.00 | 3.20 | 3.79 |
| RAL_371 | 22.55 | 20.50 | 15.12 | 13.90 | 4.53 | 6.11 | 4.55 | 6.57 |
| RAL_373 | 11.05 | 10.27 | 10.95 | 11.67 | 1.83 | 2.57 | 0.37 | 3.07 |
| RAL_374 | 14.32 | 11.54 | 12.00 | 12.03 | 4.96 | 7.75 | 5.43 | 7.69 |
| RAL_375 | 12.70 | 12.85 | 5.28 | 7.60 | 4.14 | 6.00 | 4.14 | 3.23 |
| RAL_377 | 27.85 | 25.10 | 21.32 | 24.45 | 6.52 | 11.56 | 9.42 | 10.66 |
| RAL_378 | 19.78 | 15.64 | 23.88 | 23.95 | 4.03 | 3.34 | 5.07 | 6.77 |
| RAL_379 | 16.80 | 17.49 | 6.96 | 8.89 | 2.76 | 2.64 | 2.92 | 3.74 |
| RAL_380 | 10.00 | 7.59 | 10.56 | 10.88 | 2.23 | 2.28 | 1.97 | 1.50 |
| RAL_381 | 13.53 | 12.79 | 11.05 | 10.04 | 4.81 | 5.27 | 2.72 | 2.97 |
| RAL_382 | 13.46 | 14.41 | 13.32 | 12.83 | 5.60 | 7.29 | 4.43 | 6.93 |
| RAL_383 | 19.00 | 17.69 | 16.59 | 19.14 | 4.68 | 5.37 | 4.93 | 7.03 |
| RAL_385 | 17.75 | 20.46 | 14.83 | 16.85 | 5.52 | 9.23 | 4.87 | 7.09 |
| RAL_386 | 20.71 | 20.85 | 15.87 | 16.18 | 5.90 | 8.84 | 8.30 | 9.07 |
| RAL_387 | 18.68 | 22.50 | 14.99 | 16.40 | 5.53 | 11.87 | 4.67 | 10.10 |
| RAL_391 | 17.43 | 17.28 | 14.01 | 20.57 | 4.10 | 5.39 | 3.35 | 8.50 |
| RAL_392 | 19.75 | 19.86 | 16.42 | 17.81 | 9.18 | 14.89 | 9.14 | 14.11 |
| RAL_398 | 17.98 | 17.04 | 16.22 | 14.52 | 3.55 | 3.33 | 6.48 | 4.07 |
| RAL_399 | 23.29 | 22.75 | 14.46 | 19.28 | 8.04 | 10.00 | 3.14 | 6.30 |
| RAL_405 | 13.88 | 14.54 | 11.39 | 13.28 | 5.87 | 6.36 | 3.52 | 4.52 |
| RAL_406 | 8.63 | 9.24 | 9.67 | 10.27 | 5.00 | 5.79 | 1.93 | 3.89 |
| RAL_409 | 9.64 | 10.48 | 11.07 | 11.48 | 3.41 | 2.88 | 3.71 | 6.09 |
| RAL_426 | 12.38 | 15.52 | 11.77 | 15.07 | 1.68 | 1.42 | 1.53 | 3.61 |
| RAL_427 | 13.27 | 15.91 | 7.11 | 10.34 | 0.62 | 4.79 | 1.50 | 2.71 |
| RAL_437 | 19.07 | 23.51 | 14.49 | 16.90 | 4.20 | 4.36 | 6.40 | 4.81 |
| RAL_439 | 16.75 | 16.94 | 15.17 | 15.40 | 4.45 | 7.80 | 2.97 | 4.10 |
| RAL_440 | 18.15 | 17.80 | 15.42 | 13.97 | 5.83 | 7.47 | 5.45 | 5.59 |
| RAL_441 | 12.32 | 14.52 | 13.17 | 14.59 | 3.97 | 5.59 | 3.29 | 3.57 |
| RAL_443 | 17.38 | 17.74 | 13.62 | 17.07 | 2.69 | 4.79 | 3.79 | 5.23 |
| RAL_461 | 13.94 | 18.07 | 11.25 | 13.24 | 0.83 | 5.36 | 1.00 | 4.22 |
| RAL_476 | 17.62 | 18.21 | 15.08 | 15.20 | 3.39 | 1.97 | 3.53 | 3.30 |
| RAL_486 | 13.76 | 13.46 | 6.45 | 13.11 | 3.89 | 3.00 | 2.97 | 4.90 |
| RAL_491 | 10.18 | 9.65 | 10.92 | 10.17 | 1.63 | 3.23 | 2.13 | 4.23 |
| RAL_492 | 10.23 | 12.16 | 9.74 | 11.53 | 0.41 | 2.30 | 0.53 | 1.50 |
| RAL_502 | 14.44 | 13.79 | 15.54 | 15.29 | 3.38 | 6.20 | 6.38 | 4.87 |
| RAL_508 | 12.48 | 13.35 | 14.00 | 12.20 | 1.48 | 2.59 | 2.61 | 2.17 |
| RAL_509 | 11.58 | 14.99 | 12.40 | 10.81 | 8.40 | 10.60 | 6.40 | 7.03 |
| RAL_513 | 8.98 | 10.04 | 10.65 | 11.18 | 1.50 | 2.10 | 1.97 | 2.71 |
| RAL_514 | 19.06 | 21.71 | 18.86 | 20.91 | 9.77 | 12.52 | 5.46 | 7.74 |
| RAL_517 | 11.47 | 9.48 | 8.07 | 7.81 | 4.27 | 4.31 | 3.10 | 2.86 |
| RAL_530 | 15.58 | 19.24 | 14.54 | 14.45 | 6.48 | 7.76 | 3.39 | 5.11 |
| RAL_531 | 11.37 | 14.29 | 13.84 | 16.63 | 1.68 | 4.52 | 1.75 | 3.90 |
| RAL_535 | 11.82 | 11.27 | 11.18 | 11.90 | 4.20 | 8.20 | 5.48 | 5.62 |
| RAL_554 | 16.45 | 16.80 | 15.24 | 19.49 | 3.83 | 3.90 | 4.00 | 4.26 |
| RAL_555 | 14.77 | 15.44 | 11.52 | 18.44 | 4.50 | 6.90 | 3.87 | 4.09 |
| RAL_563 | 30.80 | 29.21 | 25.22 | 27.20 | 11.63 | 12.31 | 9.77 | 13.31 |
| RAL_584 | 26.25 | 28.24 | 19.83 | 19.87 | 4.13 | 8.34 | 6.43 | 8.70 |
| RAL_589 | 10.81 | 11.44 | 13.35 | 12.58 | 1.81 | 3.79 | 0.77 | 3.63 |
| RAL_595 | 17.04 | 16.65 | 11.62 | 14.77 | 2.42 | 4.19 | 3.23 | 4.38 |
| RAL_639 | 13.24 | 13.32 | 8.81 | 8.93 | 3.80 | 4.17 | 3.47 | 6.33 |
| RAL_642 | 16.48 | 17.07 | 14.42 | 14.40 | 3.83 | 5.47 | 4.28 | 5.53 |
| RAL_646 | 17.92 | 14.69 | 13.80 | 16.72 | 3.19 | 1.97 | 2.85 | 5.06 |
| RAL_703 | 11.65 | 15.58 | 12.18 | 16.02 | 3.33 | 3.00 | 2.50 | 3.39 |
| RAL_705 | 8.57 | 11.81 | 7.51 | 10.01 | 4.74 | 7.79 | 2.87 | 7.44 |
| RAL_707 | 13.61 | 14.34 | 13.31 | 13.64 | 9.90 | 13.23 | 3.26 | 3.45 |
| RAL_712 | 17.27 | 15.67 | 21.00 | 29.27 | 3.97 | 4.90 | 4.69 | 10.47 |
| RAL_714 | 20.50 | 21.82 | 14.60 | 24.93 | 6.41 | 10.37 | 6.96 | 9.79 |
| RAL_716 | 10.92 | 14.37 | 11.57 | 14.08 | 3.55 | 8.83 | 4.41 | 8.63 |
| RAL_721 | 14.20 | 15.55 | 13.44 | 14.39 | 6.84 | 8.50 | 5.90 | 5.93 |
| RAL_727 | 8.82 | 9.26 | 10.68 | 11.98 | 3.55 | 3.83 | 3.03 | 2.24 |
| RAL_730 | 12.03 | 13.91 | 6.31 | 8.55 | 2.87 | 3.28 | 3.00 | 6.71 |
| RAL_732 | 13.63 | 15.42 | 16.28 | 15.00 | 6.14 | 8.48 | 9.41 | 7.45 |
| RAL_737 | 21.35 | 21.17 | 14.15 | 16.16 | 9.82 | 11.30 | 8.69 | 10.45 |
| RAL_738 | 20.72 | 19.25 | 12.34 | 15.08 | 3.38 | 6.90 | 4.53 | 5.90 |
| RAL_748 | 17.48 | 19.81 | 14.95 | 17.15 | 4.71 | 7.97 | 6.23 | 5.53 |
| RAL_757 | 13.89 | 11.27 | 11.27 | 11.66 | 1.86 | 3.27 | 3.62 | 5.00 |
| RAL_761 | 16.85 | 21.00 | 16.77 | 21.97 | 4.08 | 5.94 | 5.17 | 6.74 |
| RAL_765 | 7.66 | 9.32 | 7.96 | 10.33 | 1.36 | 2.50 | 2.74 | 2.86 |
| RAL_774 | 30.33 | 29.79 | 21.45 | 25.45 | 7.54 | 6.21 | 5.52 | 5.77 |
| RAL_776 | 12.83 | 14.54 | 12.74 | 15.33 | 2.00 | 4.00 | 2.54 | 4.68 |
| RAL_783 | 13.27 | 16.74 | 14.24 | 15.65 | 3.73 | 5.44 | 4.40 | 6.90 |
| RAL_786 | 13.00 | 12.39 | 7.95 | 6.10 | 6.30 | 6.30 | 4.90 | 5.39 |
| RAL_787 | 9.12 | 10.37 | 11.38 | 8.95 | 2.74 | 4.77 | 2.97 | 4.69 |
| RAL_790 | 10.78 | 12.92 | 10.15 | 14.11 | 4.13 | 3.87 | 3.20 | 3.45 |
| RAL_796 | 14.93 | 14.08 | 11.10 | 15.49 | 3.44 | 8.00 | 3.72 | 6.43 |
| RAL_799 | 14.83 | 15.15 | 15.23 | 23.86 | 5.86 | 10.43 | 6.80 | 11.23 |
| RAL_801 | 15.88 | 15.90 | 9.62 | 12.37 | 2.93 | 4.20 | 1.76 | 3.14 |
| RAL_802 | 20.13 | 19.28 | 18.05 | 15.10 | 8.23 | 9.28 | 6.60 | 6.20 |
| RAL_804 | 14.04 | 14.09 | 13.01 | 12.32 | 4.71 | 3.45 | 2.87 | 6.43 |
| RAL_805 | 10.65 | 12.93 | 9.34 | 11.34 | 3.70 | 5.03 | 2.17 | 4.43 |
| RAL_808 | 16.02 | 18.30 | 11.81 | 13.26 | 3.50 | 6.10 | 1.45 | 1.38 |
| RAL_810 | 19.34 | 21.48 | 13.37 | 15.92 | 5.77 | 13.50 | 3.72 | 4.93 |
| RAL_812 | 12.12 | 13.14 | 11.48 | 13.77 | 3.52 | 3.77 | 2.93 | 7.75 |
| RAL_818 | 19.05 | 23.64 | 14.54 | 16.81 | 4.17 | 6.67 | 2.83 | 8.87 |
| RAL_819 | 21.43 | 23.73 | 16.06 | 18.96 | 5.23 | 10.41 | 7.52 | 8.77 |
| RAL_820 | 12.90 | 14.76 | 17.76 | 10.99 | 5.10 | 5.31 | 2.93 | 4.63 |
| RAL_821 | 10.35 | 13.27 | 10.90 | 12.56 | 2.97 | 6.26 | 1.69 | 3.44 |
| RAL_822 | 22.40 | 21.94 | 20.30 | 20.58 | 8.03 | 9.10 | 11.21 | 10.87 |
| RAL_832 | 14.39 | 19.38 | 14.95 | 17.41 | 7.26 | 8.78 | 4.54 | 5.68 |
| RAL_837 | 19.48 | 22.17 | 19.46 | 22.62 | 9.17 | 14.97 | 8.33 | 11.77 |
| RAL_843 | 11.40 | 12.16 | 12.36 | 12.54 | 3.80 | 2.79 | 3.50 | 3.45 |
| RAL_849 | 17.86 | 14.49 | 15.89 | 14.84 | 3.17 | 4.59 | 3.22 | 3.47 |
| RAL_850 | 15.14 | 16.79 | 13.69 | 14.12 | 5.73 | 8.16 | 4.59 | 7.19 |
| RAL_852 | 25.94 | 26.35 | 24.08 | 25.66 | 13.97 | 14.77 | 10.41 | 8.76 |
| RAL_853 | 11.86 | 13.86 | 11.93 | 11.87 | 2.82 | 3.89 | 4.34 | 4.20 |
| RAL_855 | 16.52 | 18.34 | 12.95 | 11.25 | 5.90 | 8.41 | 5.57 | 8.26 |
| RAL_857 | 23.45 | 22.93 | 14.89 | 16.24 | 5.00 | 4.94 | 5.00 | 9.00 |
| RAL_859 | 13.89 | 13.96 | 16.28 | 13.77 | 6.29 | 12.45 | 6.69 | 7.44 |
| RAL_861 | 11.43 | 11.35 | 13.95 | 16.31 | 6.13 | 5.10 | 4.55 | 6.68 |
| RAL_879 | 14.93 | 14.01 | 13.84 | 12.81 | 1.37 | 4.00 | 1.55 | 2.23 |
| RAL_882 | 20.70 | 19.71 | 11.82 | 14.81 | 8.47 | 10.69 | 2.60 | 11.07 |
| RAL_884 | 13.61 | 14.91 | 10.87 | 12.68 | 4.27 | 4.79 | 5.55 | 5.42 |
| RAL_887 | 10.50 | 12.66 | 10.21 | 11.69 | 1.38 | 4.25 | 1.64 | 2.23 |
| RAL_890 | 19.34 | 16.35 | 17.84 | 15.02 | 3.33 | 3.79 | 2.87 | 3.70 |
| RAL_892 | 17.12 | 17.18 | 11.30 | 11.35 | 1.67 | 6.00 | 1.16 | 3.07 |
| RAL_894 | 21.48 | 22.82 | 14.97 | 17.54 | 10.55 | 12.03 | 7.61 | 7.39 |
| RAL_897 | 24.28 | 21.48 | 14.44 | 15.33 | 4.21 | 5.50 | 1.96 | 3.18 |
| RAL_900 | 17.48 | 19.20 | 11.32 | 16.02 | 4.52 | 4.34 | 1.43 | 4.70 |
| RAL_907 | 14.20 | 14.44 | 10.79 | 10.28 | 2.32 | 2.50 | 2.00 | 2.97 |
| RAL_908 | 13.98 | 12.17 | 10.60 | 10.82 | 3.80 | 6.87 | 2.97 | 3.33 |
| RAL_911 | 13.77 | 15.34 | 16.34 | 15.93 | 2.59 | 4.17 | 4.00 | 6.53 |
| RAL_913 | 20.42 | 21.47 | 13.30 | 16.20 | 12.10 | 11.57 | 9.40 | 13.66 |

Mean phenotypic values by sex for Startle Response (SR) and Negative Geotaxis (NG), of control (C) treatment or 3mM Menadione sodium bisulfite (MSB) treatment. *Raw data corrected for block effect.
