# Supplementary material for: Genome-Wide Association for Sensitivity to Chronic Oxidative Stress in Drosophila melanogaster
Source: PLoS One. 2012 Jun 8;7(6):e38722. doi: 10.1371/journal.pone.0038722 (PMC3371005; doi:10.1371/journal.pone.0038722)
Supplement: Table S2 — ANOVA of locomotor traits across treatments. (DOCX) [file pone.0038722.s007.docx]

**Supplementary Table 2**

**ANOVA of locomotor traits across treatments**

| **Trait** | **Source** | **df** | **MS** | **F** | ***P*** | ***σ*^2^** |
| --- | --- | --- | --- | --- | --- | --- |
| **Startle Response** | Block (B) | 11 | 3696.54 | 11.63 | <0.0001 | 11.24 |
|  | Treatment (T) | 1 | 12715 | 53.25 | <0.0001 | Fixed |
|  | Sex (L) | 1 | 3453.68 | 60.02 | <0.0001 | Fixed |
|  | L(B) | 363 | 1047.24 | 4.22 | <0.0001 | 15.62 |
|  | S×L(B) | 261 | 66.14 | 1.35 | 0.0083 | 0 |
|  | T×S | 1 | 63.94 | 1.43 | 0.2326 | Fixed |
|  | T×L(B) | 203 | 321.71 | 6.87 | <0.0001 | 6.64 |
|  | S×T×L(B) | 188 | 46.74 | 0.49 | 1.0000 | 0.53 |
|  | Replicate(S×T×L×B) | 689 | 100.07 | 3.79 | <0.0001 | 2.64 |
|  | Error | 21409 | 26.43 |  |  | 27.33 |
| **Negative Geotaxis** | Block (B) | 11 | 192.06 | 1.02 | 0.4305 | 0.016 |
|  | Treatment (T) | 1 | 499.94 | 7.21 | 0.0077 | Fixed |
|  | Sex (L) | 1 | 8895.52 | 174.86 | <0.0001 | Fixed |
|  | L(B) | 339 | 329.95 | 3.76 | <0.0001 | 4.21 |
|  | S×L(B) | 267 | 59.79 | 1.54 | 0.0005 | 0.33 |
|  | T×S | 1 | 65.31 | 1.71 | 0.1918 | Fixed |
|  | T×L(B) | 198 | 81.37 | 2.10 | <0.0001 | 1.10 |
|  | S×T×L(B) | 178 | 38.87 | 0.77 | 0.9826 | 0.29 |
|  | Replicate(S×T×L×B) | 696 | 51.62 | 1.65 | <0.0001 | 0.69 |
|  | Error | 20581 | 31.26 |  |  | 31.62 |

df: degrees of freedom; MS: Type III Mean Squares; F: F-statistic; *P*: *P*-value; *σ*^2^: Variance component.
