# Supplementary material for: Genome-Wide Association for Sensitivity to Chronic Oxidative Stress in Drosophila melanogaster
Source: PLoS One. 2012 Jun 8;7(6):e38722. doi: 10.1371/journal.pone.0038722 (PMC3371005; doi:10.1371/journal.pone.0038722)
Supplement: Table S3 — ANOVA of locomotor traits within treatments. (DOCX) [file pone.0038722.s008.docx]

**Supplementary Table 3**

**ANOVA of locomotor traits within treatments**

| **Trait, Environment** | **Analysis** | **Source** | **df** | **MS** | **F** | ***P*** | ***σ*^2^** |
| --- | --- | --- | --- | --- | --- | --- | --- |
| **Startle,** | Sexes Pooled | Line (L) | 191 | 1298.66 | 24.77 | <0.0001 | 19.91 |
| **Control*** |  | Sex (S) | 1 | 1787.18 | 34.15 | <0.0001 | Fixed |
|  |  | L×S | 191 | 52.43 | 0.37 | 1.0000 | 0 |
|  |  | Replicate(L×S) | 384 | 141.73 | 5.00 | <0.0001 | 5.58 |
|  |  | Error | 10783 | 28.33 |  |  | 28.33 |
|  | Female | Line (L) | 191 | 689.24 | 5.13 | <0.0001 | 18.63 |
|  |  | Replicate(L) | 192 | 134.41 | 4.84 | <0.0001 | 7.13 |
|  |  | Error | 5384 | 27.77 |  |  | 27.77 |
|  | Male | Line (L) | 191 | 662.62 | 4.45 | <0.0001 | 17.20 |
|  |  | Replicate(Line) | 192 | 149.04 | 5.16 | <0.0001 | 8.02 |
|  |  | Error | 5399 | 28.90 |  |  | 28.90 |
| **Startle,** | Sexes Pooled | Line (L) | 191 | 951.55 | 9.41 | <0.0001 | 14.13 |
| **Menadione*** |  | Sex (S) | 1 | 2817.26 | 28.17 | <0.0001 | Fixed |
|  |  | L×S | 191 | 101.13 | 1.29 | 0.0205 | 0.73 |
|  |  | Replicate(L×S) | 384 | 78.96 | 3.10 | <0.0001 | 3.57 |
|  |  | Error | 10810 | 25.47 |  |  | 25.46 |
|  | Female | Line (L) | 191 | 475.60 | 6.07 | <0.0001 | 13.27 |
|  |  | Replicate(L) | 192 | 78.31 | 3.23 | <0.0001 | 3.62 |
|  |  | Error | 5384 |  |  |  | 24.27 |
|  | Male | Line (L) | 191 | 590.34 | 7.42 | <0.0001 | 16.40 |
|  |  | Replicate(Line) | 192 | 79.61 | 2.99 | <0.0001 | 3.52 |
|  |  | Error | 5399 |  |  |  | 26.66 |
| **Geotaxis,** | Sexes Pooled | Line (L) | 191 | 369.70 | 6.20 | <0.0001 | 5.33 |
| **Control** |  | Sex (S) | 1 | 8797.15 | 147.95 | <0.0001 | Fixed |
|  |  | L×S | 191 | 59.58 | 1.00 | 0.5094 | 0 |
|  |  | Replicate(L×S) | 384 | 59.90 | 1.81 | <0.0001 | 1.84 |
|  |  | Error | 10429 | 33.02 |  |  | 33.02 |
|  | Females | Line (L) | 191 | 164.84 | 3.46 | <0.0001 | 4.04 |
|  |  | Replicate(L) | 192 | 47.66 | 1.90 | <0.0001 | 1.55 |
|  |  | Error | 5197 | 25.14 |  |  | 25.15 |
|  | Males | Line (L) | 191 | 256.91 | 3.66 | <0.0001 | 6.38 |
|  |  | Replicate(Line) | 192 | 70.27 | 1.87 | <0.0001 | 2.24 |
|  |  | Error | 5232 | 37.56 |  |  | 37.56 |
| **Geotaxis,** | Sexes Pooled | Line (L) | 191 | 330.27 | 5.88 | <0.0001 | 4.75 |
| **Menadione** |  | Sex (S) | 1 | 7208.37 | 132.55 | <0.0001 | Fixed |
|  |  | L×S | 191 | 56.18 | 1.18 | 0.0930 | 0.30 |
|  |  | Replicate(L×S) | 382 | 47.94 | 1.61 | <0.0001 | 1.25 |
|  |  | Error | 10314 | 29.75 |  |  | 29.75 |
|  | Females | Line (L) | 191 | 150.80 | 3.96 | <0.0001 | 3.90 |
|  |  | Replicate(L) | 191 | 38.13 | 1.62 | <0.0001 | 1.00 |
|  |  | Error | 5162 | 23.57 |  |  | 23.57 |
|  | Males | Line (L) | 191 | 256.91 | 3.66 | <0.0001 | 6.24 |
|  |  | Replicate(Line) | 192 | 70.27 | 1.87 | <0.0001 | 1.50 |
|  |  | Error | 5232 | 37.56 |  |  | 35.94 |

df: degrees of freedom; MS: Type III Mean Squares; F: F-statistic; *P*: *P*-value; *σ*^2^: Variance component.

*Startle response data are corrected for block effect as described previously.
