# Supplementary material for: Genome-Wide Association for Sensitivity to Chronic Oxidative Stress in Drosophila melanogaster
Source: PLoS One. 2012 Jun 8;7(6):e38722. doi: 10.1371/journal.pone.0038722 (PMC3371005; doi:10.1371/journal.pone.0038722)
Supplement: Table S4 — Quantitative genetic analysis of sensitivity. (DOCX) [file pone.0038722.s009.docx]

**Supplementary Table 4**

**Quantitative genetic analysis of sensitivity**

| **A** | **Analysis** | ${\bar{\boldsymbol{X}}}_{\boldsymbol{C}}$ | ${\bar{\boldsymbol{X}}}_{\boldsymbol{T}}$ | | ***cov_CT_*** | | ***r_P_*** | | ***σ_M_^2^*** | | ***σ_S_^2^*** | | ***cov_MS_*** | | ***r_MS_*** | | ***r_GE_*** | |  |
| --- | --- | --- | --- | --- | --- | --- | --- | --- | --- | --- | --- | --- | --- | --- | --- | --- | --- | --- | --- |
|  | **Startle** | 15.93 | | 14.00 | | 13.81 | | 0.74 | | 15.4 | | 1.74 | | 1.50 | | 0.29 | | 0.82 | |
|  | **Geotaxis** | 5.39 | | 4.98 | | 4.33 | | 0.72 | | 4.59 | | 10.83 | | 0.71 | | 0.10 | | 0.82 | |
|  |  |  | |  | |  | |  | |  | |  | |  | |  | |  | |
| **B** | **Environment** | ${\bar{\boldsymbol{X}}}_{\boldsymbol{S}}$ | | ${\bar{\boldsymbol{X}}}_{\boldsymbol{G}}$ | | ***Cov_SG_*** | | ***r_P_*** | | ***σ_M_^2^*** | | ***σ_S_^2^*** | | ***cov_MS_*** | | ***r_MS_*** | | ***r_G_*** | |
|  | **Control** | 15.93 | | 5.39 | | 6.20 | | 0.53 | | 9.43 | | 0.12 | | 0.69 | | 0.66 | | 0.60 | |
|  | **MSB** | 14.00 | | 4.98 | | 4.80 | | 0.50 | | 6.98 | | 0.12 | | 0.52 | | 0.57 | | 0.55 | |

(A) $\bar{X}_{C}$**,** overall mean of control environment; $\bar{X}_{T}$**,** overall mean of MSB treatment; *cov_CT_*, covariance between line means in control and MSB environments; ***r_P_***, Pearson phenotypic correlation between control and MSB treatment environments; *σ_M_^2^*, variance of means; *σ_S_^2^* variance of sensitivities; *cov_MS_*, covariance between means and sensitivities; *r_MS_*, correlations between means and sensitivities; *r_GE_*, cross-environment genetic correlation.

(B) $\bar{X}_{S}$**,** overall mean of startle response; $\bar{X}_{T}$**,** overall mean of geotaxis; *cov_SG_*, covariance between line means of startle response and geotaxis; ***r_P_***, Pearson phenotypic correlation between startle response and geotaxis; *σ_M_^2^*, variance of means; *σ_S_^2^* variance of sensitivities; *cov_MS_*, covariance between means and sensitivities; *r_MS_*, correlations between means and sensitivities; *r_GE_*, cross-trait genetic correlation.
