# Supplementary material for: Genome-Wide Association for Sensitivity to Chronic Oxidative Stress in Drosophila melanogaster
Source: PLoS One. 2012 Jun 8;7(6):e38722. doi: 10.1371/journal.pone.0038722 (PMC3371005; doi:10.1371/journal.pone.0038722)
Supplement: Table S6 — Overlap between SNPs and genes from GWA analysis. (DOCX) [file pone.0038722.s011.docx]

**Supplementary Table 6**

**Overlap between SNPs and genes from GWA analysis**

| **Behaviors** | **SNP Label (Gene)** | **Gene Names** |
| --- | --- | --- |
| Startle Response MSB,  Startle Response Sensitivity | *2L_2954678 (lilli)*  *2L_2954687 (lilli)*  *3R_9943734 (Kif19A)*  *X_15719878 (CG8509 and Chc)*  *X_17622797 (CG42684)*  *X_3165776 (Sgs4)*  *X_4892393 (CG12680)* | *CG10793*, *CG12432*, *CG12672*, *CG12680*, *CG13218*, *CG31773*, *CG34176*, *CG3921*, *CG42684*, *CG6867*, *CG8509*, *Chc*, *dnc*, *ed*, *kek5*, *Kif19A*, *l(2)k16918*, *Lar*, *lilli*, *Msp-300*, *Mur2B*, *ovo*, *ppk23*, *Rbp9*, *salm*, *Sgs4*, *Sh*, *Socs16D* |
| Negative Geotaxis MSB, Negative Geotaxis Sensitivity | *3L_5798566 (S6k)* | *CG3631*, *CG42272*, *CG7458*, *CG8177*, *esg*, *kek3*, *lace*, *nht*, *Or35a*, *pdm3*, *rols*, *S6k*, *Sema-1a* |
| Startle Response MSB, Negative Geotaxis MSB | *2L_3056210 (CG3523)*  *2L_3969848 (CG31773)*  *2L_3969859 (CG31773)*  *3L_3919520 (Eip63F-1)* | *CG2772*,*CG31773*, *CG31952*, *CG33298*, *CG34176*, *CG3523*, *CG3921*, *CG42313*, *ed*, *Eip63F-1*, *lilli*, *salm*, *Sema-1a*, *snRNA:U5:23D*, *sob* |
| Startle Response Sensitivity, Negative Geotaxis Sensitivity |  | *CG12672*, *CG14317*, *CG32365*, *CG42268*, *CG42340*, *CG6867*, *dpr12*, *His1:CG33801*, *His-Psi:CR31754*, *jing*, *Lar*, *mirr*, *Sh* |
| Startle Response Sensitivity, Negative Geotaxis Sensitivity, Startle MSB |  | *CG12672*, *CG6867*, *Lar*, *Sh* |
