# Supplementary material for: Genome-Wide Association for Sensitivity to Chronic Oxidative Stress in Drosophila melanogaster
Source: PLoS One. 2012 Jun 8;7(6):e38722. doi: 10.1371/journal.pone.0038722 (PMC3371005; doi:10.1371/journal.pone.0038722)
Supplement: Table S7 — Gene-centered prediction models. (DOCX) [file pone.0038722.s012.docx]

**Supplementary Table 7**

**Gene-centered prediction models**

| **Trait** | **Analysis** | **Variant** | **SNP Location** | **Estimate** | ***t*** | ***P*** |
| --- | --- | --- | --- | --- | --- | --- |
|  |  | Intercept |  | 12.185 | 35.49 | <0.0001 |
|  |  | *X_8897627* | *CG10962* (in) | 2.531 | 4.68 | <0.0001 |
|  |  | *X_13065321* | *CG34411* (in) | 2.340 | 2.85 | 0.0049 |
|  | Females | *X_17609996* | *CG42684* (in) | 1.241 | 2.88 | 0.0045 |
|  | *r*^2^ = 0.581 | *2L_9512179* | *CG33298* (in) | 3.051 | 4.19 | <0.0001 |
|  |  | *2R_9330134* | *cnn* (in) | 2.379 | 3.74 | 0.0003 |
|  |  | *3L_3919520* | *Eip63F-1* (in) | 0.888 | 2.57 | 0.0111 |
|  |  | *3L_12155156* | *CG9760* (in) | 1.056 | 3.82 | 0.0002 |
|  |  | *3R_14327168* | *fru* (in) | -0.814 | -3.60 | 0.0004 |
|  |  | *3R_16803466* | *Calx* (-532) | 0.493 | 2.18 | 0.0311 |
|  |  | Intercept |  | 13.490 | 33.51 | <0.0001 |
|  |  | *X_16566918* | *CG13012* (in) | 2.511 | 5.48 | <0.0001 |
|  |  | *2L_4076929* | *ed* (in) | 1.939 | 3.83 | 0.0002 |
|  |  | *2L_8562136* | *Sema-1a* (in) | 1.208 | 4.08 | <0.0001 |
| Startle Response | Males | *2L_9512179* | *CG33298* (in) | 2.347 | 3.16 | 0.0019 |
| MSB | *r*^2^ = 0.653 | *2L_13880093* | *cenG1A* (in) | -0.985 | -3.93 | 0.0001 |
|  |  | *2R_7121198* | *CG13218* (cds) | 2.019 | 4.05 | <0.0001 |
|  |  | *2R_19490010* | *CG10332* (714) | 2.458 | 3.28 | 0.0013 |
|  |  | *3L_2391450* | *CG13800* (in) | 1.725 | 3.35 | 0.0010 |
|  |  | *3L_13264634* | *caps* (in) | 0.641 | 2.79 | 0.0059 |
|  |  | *3R_19388517* | *beat-IV* (in) | -0.712 | -3.19 | 0.0017 |
|  |  | Intercept |  | 14.441 | 36.98 | <0.0001 |
|  |  | *X_8897627* | *CG10962* (in) | 2.622 | 5.20 | <0.0001 |
|  |  | *X_13065321* | *CG34411* (in) | 2.636 | 3.76 | 0.0002 |
|  |  | *X_17747353* | *CG32557* (cds) | 1.561 | 2.69 | 0.0080 |
|  | Sexes Pooled | *X_19510159* | *CG14210* (-81) | 0.552 | 2.29 | 0.0232 |
|  | *r*^2^ = 0.634 | *2L_9512179* | *CG33298* (in) | 2.569 | 3.72 | 0.0003 |
|  |  | *2L_13866273* | *cenG1A* (in) | -0.856 | -3.38 | 0.0009 |
|  |  | *3L_4029375* | *Gr64b* (cds) | -0.843 | -4.13 | <0.0001 |
|  |  | *3L_5543328* | *CG10625* (in) | 2.574 | 3.72 | 0.0003 |
|  |  | *3R_16803466* | *Calx* (-532) | 0.540 | 2.54 | 0.0120 |
|  |  | *3R_19388517* | *beat-IV* (in) | -0.862 | -4.15 | <0.0001 |
|  |  | Intercept |  | 2.950 | 15.03 | <0.0001 |
|  |  | *X_3069931* | *CG3939* (in) | 1.115 | 3.70 | 0.0003 |
|  |  | *2L_1838286* | *CG31665* (in) | 0.730 | 4.55 | <0.0001 |
|  |  | *2L_3871003* | *Cep97* (cds) | 0.665 | 3.73 | 0.0003 |
|  | Females | *2L_4965341* | *CG34124* (u3) | 1.521 | 3.38 | 0.0009 |
|  | *r*^2^ = 0.622 | *2L_14841373* | *CG42682* (-755) | 0.388 | 3.15 | 0.0020 |
|  |  | *2L_15748807* | *l(2)35Di* (-794) | 0.450 | 2.48 | 0.0142 |
|  |  | *2R_4223620* | *pdm3* (in) | -0.455 | -3.49 | 0.0006 |
|  |  | *2R_15050503* | *ena* (379) | 0.499 | 3.02 | 0.0029 |
|  |  | *2R_18830480* | *CG42694* (-252) | 1.143 | 4.42 | <0.0001 |
|  |  | *2R_20569780* | *Mmp1* (cds) # | -1.301 | -2.57 | 0.0111 |
|  |  | *3L_3919516* | *Eip63F-1*(in) | 1.146 | 4.93 | <0.0001 |
|  |  | Intercept |  | 4.310 | 18.97 | <0.0001 |
|  |  | *2L_10409596* | *RpS27A* (in) | 0.807 | 2.92 | 0.0041 |
|  |  | *2L_12928704* | *ACXE* (cds) | 0.564 | 2.93 | 0.0039 |
|  |  | *2L_18935245* | *ssp3* (in) | 0.430 | 2.70 | 0.0077 |
|  | Males | *2L_20032312* | *sNPF* (in) | 0.813 | 3.51 | 0.0006 |
| Negative Geotaxis | *r*^2^ = 0.638 | *2R_3647319* | *CG30497* (in) | 0.761 | 2.17 | 0.0312 |
| MSB |  | *2R_11045775* | *igl* (in) | 0.673 | 4.43 | <0.0001 |
|  |  | *2R_14918476* | *5-HT1B* (in) | -0.768 | -4.58 | <0.0001 |
|  |  | *3L_3627684* | *CG12029* (in) | 1.072 | 4.36 | <0.0001 |
|  |  | *3L_9252114* | *CG42509* (in) | 1.219 | 3.27 | 0.0013 |
|  |  | *3R_20257065* | *nAcRalpha-96Aa* (in) | 0.984 | 4.30 | <0.0001 |
|  |  | Intercept |  | 3.652 | 19.54 | <0.0001 |
|  |  | *2L_3345391* | *E23* (in) | 0.574 | 2.08 | 0.0390 |
|  |  | *2L_3836765* | *slp2* (-77) | 1.190 | 3.46 | 0.0007 |
|  |  | 2L_9540877 | *CG17855* (cds) | 0.700 | 2.83 | 0.0053 |
|  | Sexes Pooled | *2L_15755675* | *l(2)35Df* (UTR) | 0.450 | 1.99 | 0.0486 |
|  | *r*^2^ = 0.483 | *2R_11045775* | *igl* (in) | 0.581 | 3.88 | 0.0002 |
|  |  | *3L_3627684* | *CG12029* (in) | 0.850 | 3.49 | 0.0006 |
|  |  | *3L_3919516* | *Eip63F-1* (in) | 0.838 | 2.92 | 0.0040 |
|  |  | *3R_16750446* | *CG31191* (in) | 0.541 | 2.47 | 0.0145 |
|  |  | Intercept |  | 1.470 | 6.64 | <0.0001 |
|  |  | *2L_6484517* | *DLP* (cds) | 0.349 | 3.75 | 0.0002 |
|  |  | *2L_20319296* | *spir* (in) | -0.300 | -3.05 | 0.0027 |
|  | Females | *2R_6039676* | *KCNQ* (in) | 0.658 | 3.93 | 0.0001 |
|  | *r*^2^ = 0.554 | *2R_6681250* | *CG33144* (in) | -0.344 | -3.07 | 0.0025 |
|  |  | *2R_19216204* | *CG30413* (-862) | -0.269 | -2.70 | 0.0078 |
|  |  | *3L_6616274* | *Glu-RI* (-860) | -0.427 | -4.47 | <0.0001 |
|  |  | *3L_7869847* | *CG32365* (in) | 0.337 | 3.42 | 0.0008 |
|  |  | *3R_8828530* | *CG7518* (cds) # | -0.749 | -4.44 | <0.0001 |
|  |  | Intercept |  | 1.626 | 8.26 | <0.0001 |
|  |  | *X_3051478* | *N* (in) | -1.039 | -3.27 | 0.0013 |
|  |  | *X_3457239* | *CG32792* (in) | -0.387 | -3.23 | 0.0015 |
|  |  | *X_16776237* | *CG4872* (cds) | 0.260 | 2.37 | 0.0188 |
|  | Males | *X_19342749* | *kek5* (in) | -0.438 | -3.20 | 0.0017 |
| Startle Response | *r*^2^ = 0.588 | *2L_20319296* | *spir* (in) | -0.289 | -2.63 | 0.0094 |
| Sensitivity |  | *2R_6681250* | *CG33144* (in) | -0.332 | -2.61 | 0.0099 |
|  |  | *2R_7120334* | *CG13218* (-435) | -0.658 | -2.27 | 0.0244 |
|  |  | *2R_17951494* | *Snp* (in) | 0.646 | 4.65 | <0.0001 |
|  |  | *2R_19789701* | *CG5591* (in) | -0.615 | -3.99 | 0.0001 |
|  |  | *3L_7362794* | *akirin* (-106) | -0.660 | -2.58 | 0.0107 |
|  |  | Intercept |  | 0.926 | 5.67 | <0.0001 |
|  |  | *X_14652453* | *CG15890* (cds) | -0.429 | -3.28 | 0.0013 |
|  |  | *2L_6484517* | *DLP* (cds) | 0.289 | 3.20 | 0.0017 |
|  |  | *2L_18083468* | *ninaD* (u3) | 0.485 | 3.93 | 0.0001 |
|  | Sexes Pooled | *2L_20319296* | *spir* (in) | -0.336 | -3.51 | 0.0006 |
|  | *r*^2^ = 0.570 | *2R_6039676* | *KCNQ* (in) | 0.571 | 3.61 | 0.0004 |
|  |  | *2R_6681251* | *CG33144* (in) | -0.297 | -2.71 | 0.0074 |
|  |  | *2R_15754475* | *Obp56i* (in) | -0.606 | -2.79 | 0.0059 |
|  |  | *2R_17951494* | *Snp* (in) | 0.556 | 4.78 | <0.0001 |
|  |  | *3L_9728206* | *CG42268* (cds) | -0.550 | -2.12 | 0.0359 |
|  |  | Intercept |  | -1.103 | -2.15 | 0.0334 |
|  |  | *X_2487674* | egh-in | -1.125 | -3.15 | 0.0019 |
|  |  | *X_8537852* | *oc* (in) | 0.878 | 3.02 | 0.0030 |
|  |  | *2L_1055671* | *S* (in) | 0.787 | 2.71 | 0.0074 |
|  | Females | *2L_8984586* | *CG13101* (in) | 1.789 | 2.87 | 0.0047 |
|  | *r*^2^ = 0.634 | *2R_17329524* | *Sdc* (in) | -1.699 | -3.41 | 0.0008 |
|  |  | *2R_18453646* | *dnr1* (cds) # | 1.643 | 4.39 | <0.0001 |
|  |  | *3L_2686716* | *CG12187* (in) | 3.195 | 5.08 | <0.0001 |
|  |  | *3L_12024281* | *rols* (in) | -1.124 | -2.84 | 0.0051 |
|  |  | *3L_21641769* | *CG33291* (in) | 0.999 | 2.97 | 0.0035 |
|  |  | *3R_6578102* | *CG14691* (-950) | 0.845 | 2.80 | 0.0058 |
|  |  | *3R_9798204* | *rdx* (in) | -2.287 | -3.39 | 0.0009 |
|  |  | Intercept |  | 0.447 | 0.90 | 0.3714 |
|  |  | *X_6396532* | *CG42340* (in) | -1.530 | -2.94 | 0.0038 |
|  |  | *2L_9193012* | *tai* (in) | 0.992 | 2.87 | 0.0047 |
|  |  | *2L_15552515* | *CG15256* (486) | 3.491 | 3.23 | 0.0015 |
|  | Males | *2R_2400146* | *jing* (in) | 2.572 | 2.95 | 0.0037 |
| Negative Geotaxis | *r*^2^ = 0.614 | *2R_8427175* | *fra* (in) | -2.147 | -3.47 | 0.0007 |
| Sensitivity |  | *2R_8484921* | *Amph* (in) | -0.893 | -2.82 | 0.0054 |
|  |  | *3L_7108114* | *form3* (in) | 3.142 | 4.37 | <0.0001 |
|  |  | *3L_12701462* | *mirr* (cds) | -1.841 | -2.75 | 0.0066 |
|  |  | *3R_8117003* | *svp* (in) | 1.445 | 4.14 | <0.0001 |
|  |  | *3R_23402905* | *wdb* (in) | 2.909 | 2.85 | 0.0050 |
|  |  | Intercept |  | 0.388 | 1.04 | 0.3022 |
|  |  | *X_13851507* | *CG11068* (900) | 1.264 | 3.15 | 0.0020 |
|  |  | *X_14300784* | *dpr8* (in) | -2.231 | -5.02 | <0.0001 |
|  |  | *2L_6964747* | *Coprox* (cds) # | 1.982 | 2.43 | 0.0163 |
|  | Sexes Pooled | *2L_21232917* | *CG31626* (cds) # | 2.045 | 3.50 | 0.0006 |
|  | *r*^2^ = 0.673 | *2R_2400146* | *jing* (in) | 2.142 | 3.40 | 0.0008 |
|  |  | *2R_17716626* | *CG13492* (cds) # | -0.979 | -3.96 | 0.0001 |
|  |  | *2R_19589537* | *CG4091* (in) | 0.743 | 2.18 | 0.0308 |
|  |  | *3L_1805960* | *CG42676* (in) | -2.063 | -3.78 | 0.0002 |
|  |  | *3L_5798566* | *CG42272* (in) | -2.319 | -3.76 | 0.0002 |
|  |  | *3L_13486761* | *CG10741* (in) | 1.713 | 4.30 | <0.0001 |
|  |  | *3L_21641769* | *CG33291* (in) | 0.887 | 2.91 | 0.0042 |
|  |  | *3R_6578102* | *CG14691* (-950) | 0.625 | 2.44 | 0.0159 |

Markers are listed in the order in which they entered the model. Estimates of effects are for (Minor allele – Major allele). In: intronic; cds: coding sequence; -/+: base pairs upstream/downstream; #: Missense; u3/5: 3’/5’ UTR.
