# Supplementary material for: Genome-Wide Association for Sensitivity to Chronic Oxidative Stress in Drosophila melanogaster
Source: PLoS One. 2012 Jun 8;7(6):e38722. doi: 10.1371/journal.pone.0038722 (PMC3371005; doi:10.1371/journal.pone.0038722)
Supplement: Table S10 — Overlap of genes in this study and GWA for acute oxidative stress. (DOCX) [file pone.0038722.s015.docx]

**Supplementary Table 10**

**Overlap of Genes in this study and GWA for acute oxidative stress**

| **Chromosome** | **Position** | **Gene Symbol** | **Flybase ID** |
| --- | --- | --- | --- |
| *2L* | 3265018 | *CG3347* | FBgn0031513 |
| *2L* | 3265018 | *CG34393* | FBgn0085422 |
| *2L* | 3302884 | *CG9663* | FBgn0031516 |
| *2L* | 3343260 | *E23* | FBgn0020445 |
| *2L* | 3733257 | *Shaw* | FBgn0003386 |
| *2L* | 3874577 | *capu* | FBgn0000256 |
| *2L* | 4076929 | *ed* | FBgn0000547 |
| *2L* | 4743467 | *CG15630* | FBgn0031627 |
| *2L* | 4899198 | *CG3036* | FBgn0031645 |
| *2L* | 6405825 | *CG42368* | FBgn0259714 |
| *2L* | 6895605 | *neuroligin* | FBgn0031866 |
| *2L* | 6895605 | *Nha1* | FBgn0031865 |
| *2L* | 6940306 | *CG18304* | FBgn0031869 |
| *2L* | 7367993 | *Wnt10* | FBgn0031903 |
| *2L* | 7529048 | *Rapgap1* | FBgn0085403 |
| *2L* | 7641085 | *CG13792* | FBgn0031927 |
| *2L* | 7641085 | *CG6739* | FBgn0031926 |
| *2L* | 9193012 | *tai* | FBgn0041092 |
| *2L* | 9475262 | *CG33723* | FBgn0053723 |
| *2L* | 9475262 | *Gdi* | FBgn0004868 |
| *2L* | 11284738 | *Osi21* | FBgn0032359 |
| *2L* | 12757521 | *CG15483* | FBgn0032457 |
| *2L* | 12757521 | *MRP* | FBgn0032456 |
| *2L* | 13866273 | *cenG1A* | FBgn0028509 |
| *2L* | 13866273 | *CG7968* | FBgn0068233 |
| *2L* | 13866273 | *Smg5* | FBgn0019890 |
| *2L* | 15893943 | *CG31735* | FBgn0051735 |
| *2L* | 15893943 | *TepI* | FBgn0041183 |
| *2L* | 15908856 | *beat-Ib* | FBgn0028645 |
| *2L* | 15917907 | *CG4793* | FBgn0028514 |
| *2L* | 15927987 | *CG12448* | FBgn0028857 |
| *2L* | 15946753 | *CG18063* | FBgn0032124 |
| *2L* | 15960666 | *CG10839* | FBgn0084636 |
| *2L* | 17864311 | *btv* | FBgn0023096 |
| *2L* | 17864311 | *CG42830* | FBgn0262017 |
| *2R* | 5412232 | *brp* | FBgn0259246 |
| *2R* | 6889468 | *luna* | FBgn0040765 |
| *2R* | 8484921 | *Amph* | FBgn0027356 |
| *2R* | 13209984 | *mbl* | FBgn0261642 |
| *2R* | 14918476 | *CG15115* | FBgn0034413 |
| *2R* | 14925940 | *CG15116* | FBgn0034415 |
| *2R* | 15050503 | *ena* | FBgn0000578 |
| *2R* | 16025952 | *CG11041* | FBgn0034481 |
| *2R* | 16328329 | *CG12484* | FBgn0086604 |
| *2R* | 16562932 | *CG30152* | FBgn0068819 |
| *2R* | 16562932 | *Gr57a* | FBgn0041240 |
| *2R* | 17693499 | *CG13492* | FBgn0034662 |
| *3L* | 2391448 | *CG42669* | FBgn0261551 |
| *3L* | 3724309 | *CG32264* | FBgn0052264 |
| *3L* | 5982409 | *CG10479* | FBgn0035656 |
| *3L* | 5982409 | *CG32406* | FBgn0052406 |
| *3L* | 8688386 | *h* | FBgn0001168 |
| *3L* | 8688386 | *SrpRbeta* | FBgn0011509 |
| *3L* | 10839382 | *tna* | FBgn0026160 |
| *3L* | 15741606 | *CG6244* | FBgn0036531 |
| *3L* | 15741606 | *comm* | FBgn0010105 |
| *3R* | 13113897 | *beat-IIa* | FBgn0038498 |
| *3R* | 13898913 | *CG14317* | FBgn0038566 |
| *3R* | 13898913 | *htl* | FBgn0010389 |
| *3R* | 13909059 | *sr* | FBgn0003499 |
| *X* | 3920374 | *CG32790* | FBgn0262973 |
| *X* | 3920374 | *CG6414* | FBgn0029690 |
| *X* | 4641269 | *CG15472* | FBgn0029724 |
| *X* | 4641269 | *Proc-R* | FBgn0029723 |
| *X* | 5180616 | *CG15464* | FBgn0029748 |
| *X* | 5180616 | *CG42749* | FBgn0261803 |
| *X* | 5406031 | *CG12730* | FBgn0029771 |
| *X* | 5744289 | *CG12729* | FBgn0029816 |
| *X* | 5744289 | *CG43136* | FBgn0262610 |
| *X* | 5862191 | *Grip* | FBgn0029830 |
| *X* | 6148601 | *Rbcn-3A* | FBgn0023458 |
| *X* | 14300784 | *betaNACtes4* | FBgn0030566 |
| *X* | 14300784 | *CG9411* | FBgn0030569 |
| *X* | 14300784 | *dpr8* | FBgn0052600 |
| *X* | 18907110 | *CG32541* | FBgn0083450 |
| *X* | 18907110 | *CG42506* | FBgn0260232 |
